# Transcriptomic profiling facilitates classification of response to influenza challenge

**DOI:** 10.1007/s00109-014-1212-8

**Published:** 2014-10-28

**Authors:** Emma E. Davenport, Richard D. Antrobus, Patrick J. Lillie, Sarah Gilbert, Julian C. Knight

**Affiliations:** 1 Wellcome Trust Centre for Human Genetics, University of Oxford, Oxford, OX3 7BN UK; 2 The Jenner Institute, University of Oxford, Oxford, OX3 7DQ UK

**Keywords:** Influenza, Challenge, Transcriptome, Expression, RNA, Microarray

## Abstract

**Abstract:**

Despite increases in vaccination coverage, reductions in influenza-related mortality have not been observed. Better vaccines are therefore required and influenza challenge studies can be used to test the efficacy of new vaccines. However, this requires the accurate post-challenge classification of subjects by outcome, which is limited in current methods that use artificial thresholds to assign ‘symptomatic’ and ‘asymptomatic’ phenotypes. We present data from an influenza challenge study in which 22 healthy adults (11 vaccinated) were inoculated with H3N2 influenza (A/Wisconsin/67/2005). We generated genome-wide gene expression data from peripheral blood taken immediately before the challenge and at 12, 24 and 48 h post-challenge. Variation in symptomatic scoring was found amongst those with laboratory confirmed influenza. By combining the dynamic transcriptomic data with the clinical parameters this variability can be reduced. We identified four subjects with severe laboratory confirmed influenza that show differential gene expression in 1103 probes 48 h post-challenge compared to the remaining subjects. We have further reduced this profile to six genes (*CCL2*, *SEPT4*, *LAMP3*, *RTP4*, *MT1G* and *OAS3*) that can be used to define these subjects. We have used this gene set to predict symptomatic infection from an independent study. This analysis gives further insight into host-pathogen interactions during influenza infection. However, the major potential value is in the clinical trial setting by providing a more quantitative method to better classify symptomatic individuals post influenza challenge.

**Key message:**

- Differential gene expression signatures are seen following influenza challenge.
- Expression of six predictive genes can classify response to influenza challenge.
- The genomic influenza response classification replicates in an independent dataset.

**Electronic supplementary material:**

The online version of this article (doi:10.1007/s00109-014-1212-8) contains supplementary material, which is available to authorized users.

## Introduction

Seasonal influenza is common, affecting 5–10 % of the 900 million people in the European Region [1] with substantial associated mortality, morbidity and socioeconomic costs. Despite increases in vaccination coverage, reductions in influenza-related mortality have not been observed [2]. In order to develop better vaccines for seasonal and pandemic influenza, influenza challenge studies are central to testing vaccine efficacy. Consenting subjects are vaccinated, quarantined and then inoculated with influenza via intranasal administration. This approach has the advantage that potentially susceptible individuals can be positively selected based on low pre-existing immunity to the challenge virus. The sample size is restricted by the capacity of quarantine facilities and the high costs associated with performing a challenge experiment. Accurate post-challenge classification of subjects by outcome is then critical to determining vaccine efficacy. However these outcomes are based on scoring systems, which have artificial thresholds for ‘symptomatic’ and ‘asymptomatic’ phenotypes and include subjective self-reported observations and on biological evidence for infection (i.e. virus shedding and post-challenge serology) that are often discordant.

We sought to investigate the utility of genome-wide gene expression profiling in classification of volunteers following challenge. Application of a transcriptomic approach in the context of a challenge study is also a powerful approach to gain new insights into the biology of disease, as concordance in the exposure history and detailed clinical phenotyping before and after infection reduce the heterogeneity inherent to study of naturally acquired infection. Gene expression microarrays have been used to characterise the aberrant nature of the immune response to the 1918 pandemic influenza virus in macaques [3]. A number of studies have applied blood transcriptomics to understand host response to influenza in the context of lower respiratory tract infection and the H1N1 pandemic [4–8]. These approaches have also been applied to the field of influenza vaccinology [9], giving insights into the mechanisms of protective immunity. There has been one previous challenge study involving H3N2 with application of transcriptomic approaches [10, 11].

Here, we describe application of gene expression profiling to an influenza challenge study involving a vaccine called MVA-NP+M1. This viral-vectored vaccine has the potential to induce heterosubtypic immunity through the induction of cytotoxic T cells with specificity for epitopes within conserved influenza proteins. We have found MVA-NP+M1 to be safe and immunogenic [12, 13] with evidence that it may increase antibody responses to inactivated influenza vaccine when both vaccines are administered together [14]. The influenza challenge study (FLU002) to test the efficacy of MVA-NP+M1 [15] showed that vaccinees with laboratory confirmed influenza (LCI) shed virus for fewer days than control subjects with LCI. In this paper, we report the nature of the transcriptional response to influenza based on genome-wide gene expression profiling of participants during the FLU002 study. We describe how this information could be used to improve the biological classification of influenza challenge subjects. These results have implications for our understanding of the host-pathogen interactions for influenza and have the potential to inform clinical diagnostic testing.

## Materials and methods

### Study subjects and clinical trial procedures

Detailed description of the subjects and methods used in the clinical trial are provided in Lillie et al. [15]. The trial protocol was approved by the local Research Ethics Committee and conducted in accordance with the Declaration of Helsinki. All participants provided written informed consent. Briefly, 22 healthy volunteers, aged 18–45 years, were enrolled and 11 were vaccinated with 1.5×10^8^ plaque forming units (pfu) of MVA-NP+M1. Thirty days after vaccination, all 22 volunteers underwent intranasal challenge with H3N2 influenza (A/Wisconsin/67/2005) at a dose of 1 ml of 10^5.25^ TCID50/ml in a quarantine facility. Volunteers were all challenged within a 2-h period. All subjects had haemagglutination inhibition (HI) titres of <1:10 to the challenge virus on admission to the quarantine unit.

After virus challenge, volunteers had a physical examination by a trial physician daily and self-reported their symptoms using a modified Jackson scoring system [16, 17] twice daily, from 12-h post-challenge, for 6 days. This system lists upper respiratory and systemic symptoms on a scale of 0–3: ‘no symptoms’, ‘just noticeable’, ‘bothersome but can still do activities’ and ‘bothersome and cannot do daily activities’. Scores were summed over the duration of the challenge period to give a total symptom score. Those volunteers with scores of ≥4 and positive viral culture from nasal wash samples were categorised as having LCI. Severity of infection was graded as mild if the summed symptom score was 4–28, with scores of ≥29 classified as moderate/severe. HI titres for each individual at 26 days after challenge are provided (Online Resource, Table S1). Volunteers were kept in quarantine until day 6 post-challenge, and all had a negative rapid antigen test on nasal wash sample prior to discharge.

Whole blood was collected in PAXgene tubes prior to influenza challenge (day 30) and then at three further time points (12, 24 and 48 h post-challenge). All nasal washing and blood collections for viral shedding and gene expression analysis were taken in the same order that the volunteers were challenged in.

### RNA extraction and globin mRNA depletion

Total RNA was extracted from whole blood using the PAXgene Blood RNA Kit (Qiagen) according to the manufacturer’s instructions. The abundance of globin mRNA present in whole blood reduces the sensitivity of expression array data, and therefore, globin mRNA depletion was carried out using the GLOBINclear^TM^-Human kit (Ambion) following the manufacturer’s instructions. Briefly, total RNA from whole blood is mixed with a biotinylated capture oligo mix specific for human globin mRNA. Streptavidin magnetic beads capture the globin mRNA. Placing the tube against a magnet then allows the globin mRNA depleted RNA to be transferred to a fresh tube. Globin-depleted RNA is further purified using a rapid, magnetic bead-based clean up procedure. Globin mRNA depleted samples were quantified using a NanoDrop spectrophotometer, and the quality for a subset was verified using a Bioanalyzer following the manufacturer’s instructions (Bioanalyzer RNA 6000 Nano kit, Agilent).

### Microarray data processing and analysis

Genome-wide gene expression analysis was carried out on 500-ng globin depleted RNA using the Illumina HumanHT-12 v4 Expression BeadChip gene expression platform comprising 47,231 probes (Core Genomics, WTCHG). The report for analysis was generated by Illumina’s GenomeStudio version 1.6.0. Background signals were subtracted and 11,158 low expression probes (detection value <0.95) were removed. The raw data were transformed and normalised using the Variance Stabilization and Normalization method [18]. Quality control checks including principal component analysis to check for batch and array effects were carried out using R [19]. Two samples were excluded as outliers. The raw and normalised data are accessible through GEO (GSE61754).

### Statistical analysis

Differential gene expression analysis was carried out on 36,073 probes using three different R packages: limma [20], maSigPro [21] and timecourse [22]. Benjamini-Hochberg corrected values are used to control for multiple testing in differential gene expression analysis and subsequent pathway analysis. Pathway and network analysis was performed using IPA (Ingenuity® Systems, www.ingenuity.com) analysing probes differentially expressed between the four moderate/severe LCI and the remaining samples, 48 h post-challenge. The pamr R package [23] was used to identify genes whose expression could accurately categorise moderate/severe LCI compared to the remaining samples. The pamr package employs a ‘nearest shrunken centroids’ method to identify subsets of genes that are best able to classify samples into the specified groups of interest. The Huang dataset [11] was normalised using the robust multi-array (RMA) method (data accessible at NCBI GEO database [24], accession GSE30550).

## Results

**Fig. 1:**
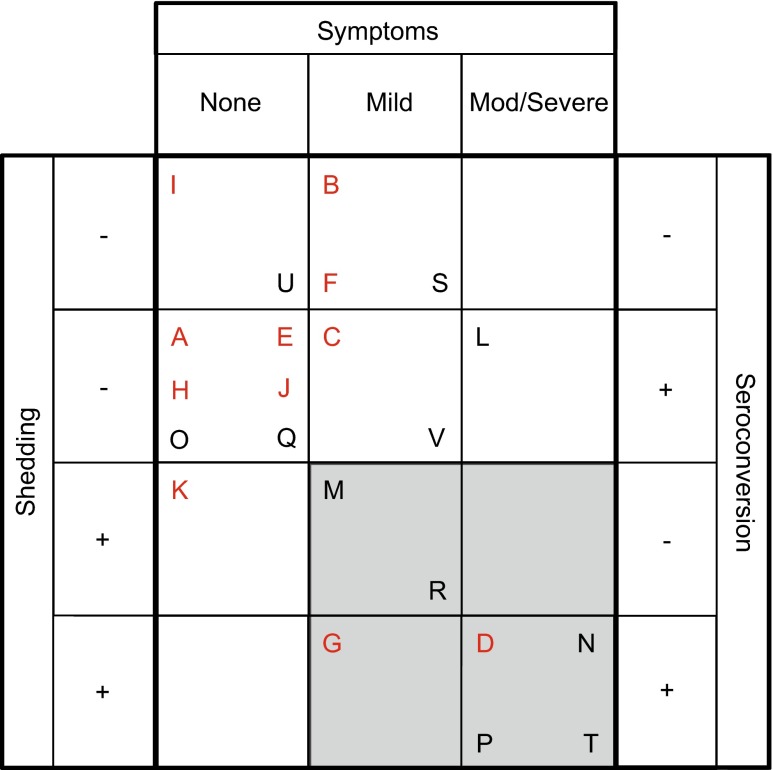
Clinical observations and classification of study subjects. Each individual is assigned a unique letter code (A-V). Vaccinated individuals are shown in *red*. Individuals are defined as having no symptoms, mild or moderate/severe based on the summed self-reported symptoms collected over 6 days. *Positive shedding* indicating viral shedding was detected on at least 1 day after challenge. Seroconversion was determined by HI titre using serum samples obtained at 26-days post-challenge (Online Resource, Table S1). LCI is defined as mild or moderate/severe symptoms and viral shedding (shown by grey shaded area)

Following influenza challenge, two individuals in the vaccinated group and five individuals in the unvaccinated group developed LCI as defined in the clinical trial protocol [15] (Fig. 1). Within the LCI group, there was considerable variation in the summed modified Jackson scores (ranging from 4 to 38) that describe the extent to which volunteers were symptomatic. We therefore hypothesised that these differences in clinical phenotype might be reflected in patterns of gene expression detected through microarray gene expression analysis.

**Fig. 2:**
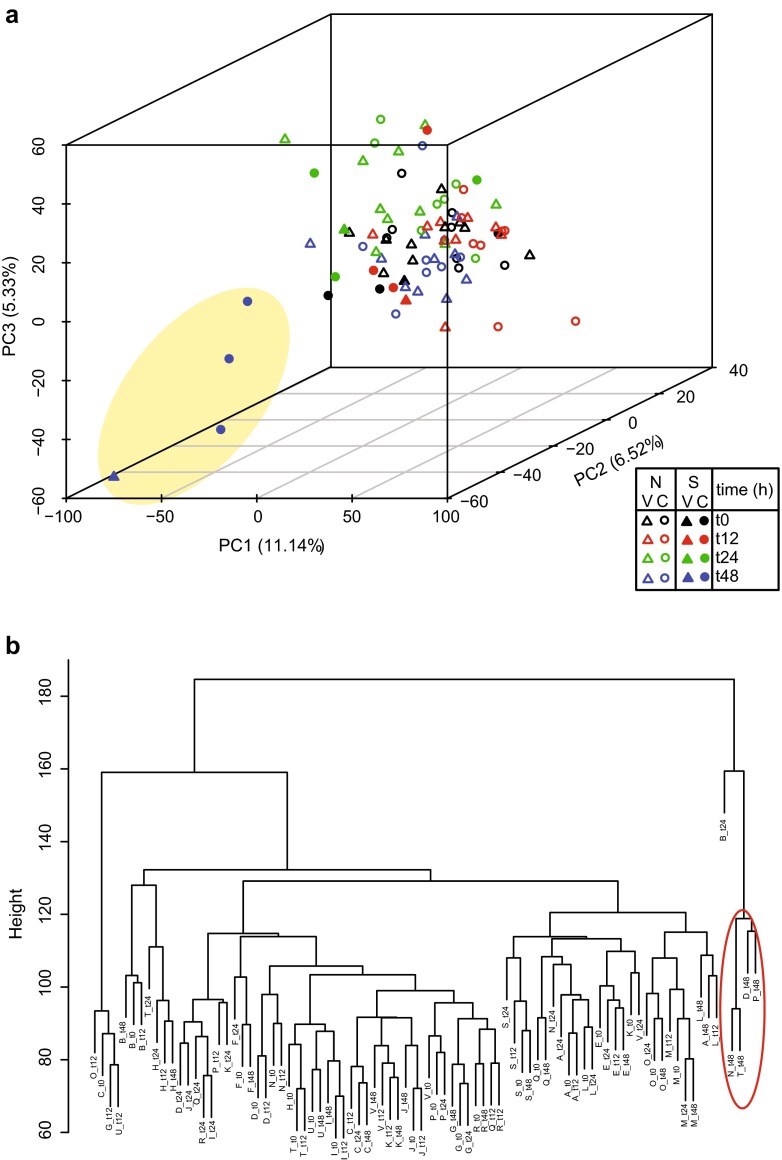
Principal components and hierarchical clustering plot. **a** The first three principal components are plotted with the proportion of variance explained by each component. The *yellow oval* highlights the samples taken from the four subjects with moderate/severe LCI 48 h post-challenge. *N* none or mild LCI, *S* moderate/severe LCI, *V* vaccinee, *C* control. **b** The hierarchical tree illustrates the relationship between clusters of samples. The *height of the branches* indicates the strength of the separation. The *red oval* highlights the samples taken from the four subjects with moderate/severe LCI 48 h post-challenge

Principal component analysis (PCA) and hierarchical clustering was performed to visualise variation in the dataset (Fig. 2). This demonstrated that four subjects shared a similar profile of variance in gene expression. These four subjects were the only cases of LCI whose symptom score was moderate/severe rather than mild. They also shared other virology outcomes as shown in Fig. 1.

We proceeded to analyse differential gene expression using a linear regression model. The likelihood of observed differences in gene expression was defined using an empirical Bayes approach in which prior distribution was estimated from the overall variance in the dataset, allowing a more stable inference given small numbers of arrays [25]. Implementing this approach using the Limma package [15], a total of 1103 probes were differentially expressed between these four volunteers and the remaining samples at 48 h post-challenge (*q* < 0.05 controlling for multiple testing using Benjamini and Hochberg’s method, Online Resource, Table S2 and Table S3). Using the same approach, no probes were differentially expressed at 12 or 24-h post-challenge (Online Resource, Table S2). This specific effect on differential expression at 48 h post-challenge is consistent with the observed temporal onset of symptoms [15].

**Fig. 3:**
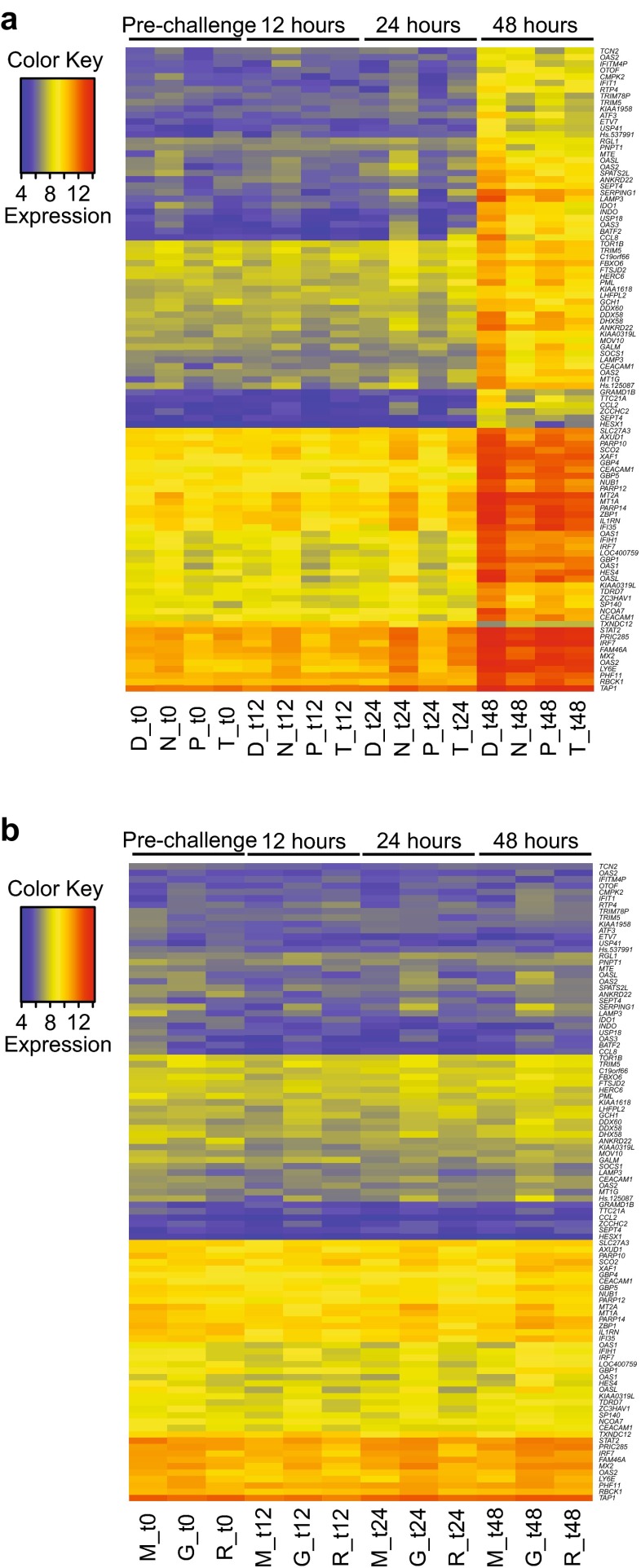
Heatmaps for differentially expressed probes. The top 100 most significant differentially expressed probes between the four moderate/severe LCI, and the remaining samples at 48 h post-challenge were used to generate heatmaps of gene expression. **a** Moderate/severe LCI subjects. **b** Mild LCI subjects. Individuals D and G were vaccinated; others are unvaccinated

In further analyses, the most significant 100 of these differentially expressed probes (9 %) were selected and almost all were found to be upregulated at 48 h post-challenge in the moderate/severe LCI individuals (Fig. 3a). In contrast, subjects with LCI and mild symptoms did not show substantial changes in expression of the same probes (Fig. 3b). To further validate these findings, two alternative methods based on a two-step regression approach (maSigPro package [21]) and on multivariate empirical Bayes statistics (timecourse package [22]) were employed to generate an equivalent list of the 200 most significant differentially expressed probes. The overlap with the original probe list was 81 and 74 %, respectively (Online Resource Fig. S1).

**Fig. 4:**
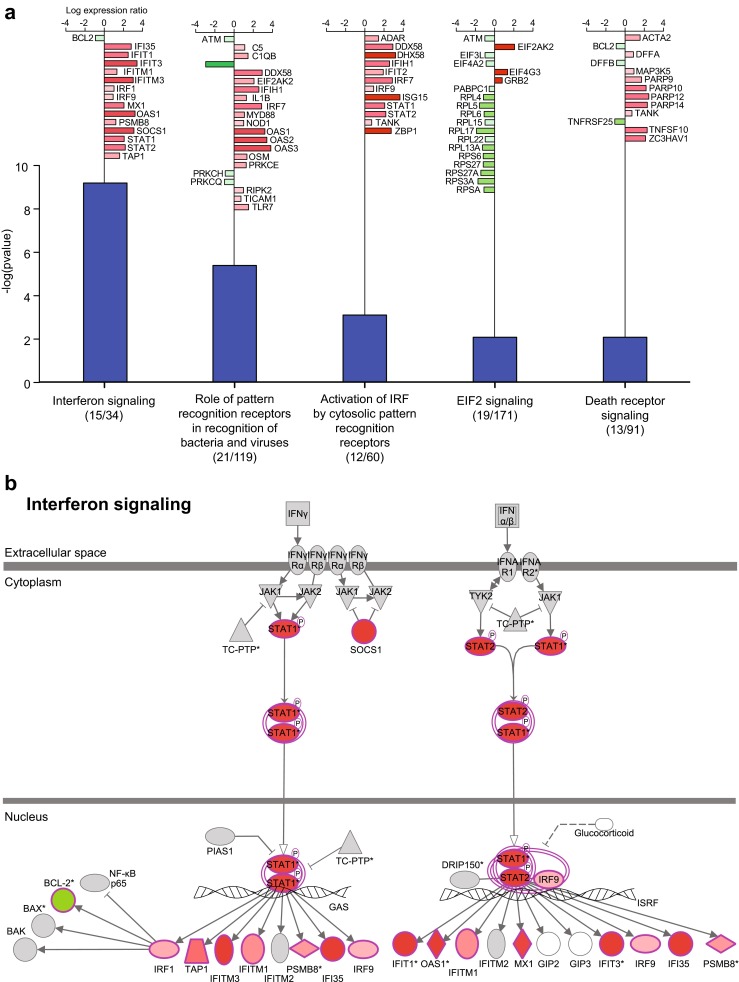
Pathway analysis of differential expression in LCI. The differentially expressed probes between the four moderate/severe LCI samples 48 h post-challenge and the remaining samples were used to determine pathway enrichment with IPA. **a** Pathways with significant enrichment based on Benjamini-Hochberg (B-H) multiple testing corrected *p* value <0.01 are shown. The *blue bars* show the −log(*p* value). Differentially expressed genes are plotted for each enriched pathway (*red* upregulated in moderate/severe LCI individuals, *green* downregulated) with numbers of enriched genes shown in *brackets* below x-axis labels. **b** The most significantly enriched pathway, interferon signalling, is shown with enriched genes shaded in colour

To assess the functional significance of the differentially expressed genes in subjects with moderate/severe LCI at 48 h post-challenge, we performed pathway and network analysis (Fig. 4, Online Resource Figure S2). This showed significant enrichment for genes involved in a number of pathways notably interferon signalling (Benjamini-Hochberg corrected *p* value = 6.4 × 10^−10^), role of pattern recognition receptors (PRR) in recognition of bacteria and viruses (*p* = 4.0 × 10^−6^), activation of IRF by cytosolic PRR (*p* = 7.7 × 10^−4^), eIF2 signalling (*p* = 8.1 × 10^−3^) and death receptor signalling (*p* = 8.1 × 10^−3^) (Fig. 4). The most significant upstream regulators identified were type III, II and I interferons (IFNL1 *p* = 2.2 × 10^−59^, IFNA2 2.7 × 10^−53^, IFNG 3.4 × 10^−42^). Network analysis further highlighted the significant upregulation of genes involved in the IFN response seen in these patients (Fig. S2). IFNs play a critical role in host defence to viral infection with the significance of IFNλ recently recognised [26–28]. The eIF2 signalling pathway plays a role in host defence by reducing translation in the host cell thereby reducing the rate at which new viral particles are generated [29] while death receptor signalling involving apoptosis has been related to disease severity in influenza A infection [30]. In terms of diseases and functions related to the differentially expressed genes at 48 h post-challenge, infectious disease was most significant, specifically replication of virus (*P* = 9.3 × 10^−17^).

Although the analysis of over 1000 differentially expressed probes can provide insight into host-pathogen interactions, this magnitude of variables does not lend itself well to use in diagnostics. We therefore sought to find the minimum number of genes that could be used to successfully classify our data. Using the tool ‘prediction analysis for microarrays’ (pamr) [23], we identified six genes (*CCL2*, *SEPT4*, *LAMP3*, *RTP4*, *MT1G* and *OAS3*) which alone classified our samples into two groups with 100 % accuracy. These two groups comprised those from subjects with moderate/severe LCI taken at 48 h post-challenge in one group and the remaining 18 samples (with either mild LCI or without LCI) in the second group. The six selected genes are strongly upregulated in individuals with moderate/severe LCI with network analysis showing relationships involving interferon activation (Online Resource Fig. S3).

In order to assess the validity of this six gene signal, we identified a data set from an independent but similarly conducted influenza challenge study, published by Huang et al. [11]. This study used similar methods for RNA collection and extraction, and the challenge was performed with the same strain of influenza (A/Wisconsin/67/2005). Of the 17 subjects in this study, eight were described as having asymptomatic infection and nine as having symptomatic infection. Those with symptomatic infection had a median summed Jackson score of 39.

For each of the subjects in the Huang study, we included six samples taken at different time points: two samples prior to challenge were included, and the remaining four samples were from consecutive post-challenge time points taken at 45, 53, 60 and 69 h. For all the post-challenge samples in the eight asymptomatic volunteers, the six gene set classified the samples with 100 % accuracy. For 89 % (8/9) of the symptomatic volunteers, pamr correctly classified at least one of these six post-challenge timepoints (Table 1).

**Table 1:** Gene signature validation. Data from the 17 subjects described by Huang et al. [11] were used to validate the predictive power of the six genes *CCL2*, *SEPT4*, *LAMP3*, *RTP4*, *MT1G* and *OAS3*

Huang I.D.	Huang phenotype	Individual sample predications by pamr						Group predicted
		Baseline sample	0 h	45 h	53 h	60 h	69 h	
1	Sx	*n*	*n*	S	S	*n*	*n*	YES
2	Asx	*n*	*n*	*n*	*n*	*n*	*n*	YES
3	Asx	*n*	*n*	*n*	*n*	n	*n*	YES
4	Asx	*n*	*n*	*n*	*n*	*n*	*n*	YES
5	Sx	*n*	*n*	S	S	S	S	YES
6	Sx	*n*	*n*	S	S	S	S	YES
7	Sx	*n*	*n*	S	S	S	n	YES
8	Sx	*n*	*n*	*n*	*n*	*S*	S	YES
9	Asx	*n*	*n*	*n*	*n*	*n*	n	YES
10	Sx	*n*	*n*	*n*	*n*	*n*	S	YES
11	Asx	*n*	*n*	*n*	*n*	*n*	n	YES
12	Sx	*n*	*n*	S	S	S	S	YES
13	Sx	–	*n*	*n*	*n*	S	S	YES
14	Asx	*n*	*n*	*n*	*n*	*n*	*n*	YES
15	Sx	*n*	*n*	*n*	*n*	*n*	*n*	NO
16	Asx	*n*	*n*	*n*	*n*	*n*	*n*	YES
17	Asx	*n*	*n*	*n*	*n*	*n*	*n*	YES

The subject number and clinical status of each subject is listed: *Sx* indicates that the subject was symptomatic and *Asx* indicates that the subject was asymptomatic. For each subject, samples were tested at six different time points. Two pre-challenge samples were tested to demonstrate that the changes in gene expression only occurred post-challenge. Four consecutive post-challenge samples were tested to cover the range over which volunteers became symptomatic. An *S* indicates that the test set sample would be classified within the moderate/severe FLU002 samples, and an *n* indicates that the samples would be classified as either a pre-challenge sample or having none/mild symptoms. With the exception of subject 15, all symptomatic subjects were correctly identified in at least one post-challenge sample

In addition, we investigated whether any differences were observed between other subgroups in the study, for instance, individuals who do not shed virus. We found no significant difference between the symptomatic and asymptomatic individuals in this subgroup. Finally, we attempted to identify whether vaccination modulated the host responses to influenza challenge. No significant difference in the temporal response to influenza was seen between vaccinees and controls on limma analysis.

## Discussion

The results of this study highlight the value of combining genome-wide gene expression profiling with clinical observations in order to improve the biological classification of influenza challenge studies and assist with clinical diagnostic testing.

Using the principal components generated from the genome-wide gene expression profiles, we were able to identify four LCI samples taken 48 h post-challenge that clustered separately from the other samples. Upon further investigation, we discovered a pattern of predominately upregulated gene expression that is not observed in the remaining samples, including LCI cases with mild symptoms according to the clinical trial protocol. The signature correlates with the clinical observations, and therefore, setting an arbitrary symptom score threshold to classify LCI may not be the most informative end result of an influenza challenge study. Instead, this gene signature may assist with redefining the symptom score threshold or could be used in combination with the symptom score to better classify moderate/severe LCI.

The informativeness of differentially expressed genes at 48 h post-challenge are likely to reflect the observed peak in influenza A viral titres that have been reported at this time for experimentally infected adults which has been shown to be closely mirrored by changes in innate immune response notably type 1 interferon levels [31, 32]. From the list of probes that we found are differentially expressed in the four moderate/severe LCI cases 48 h post-challenge, pathway analysis identified a number of pathways which are enriched within the dataset. Many of these pathways are involved in the anti-viral response illustrating that these four individuals show a different immune response to those with mild LCI. Similar pathways, and in particular, those regulated by interferon, have previously been identified in a number of other studies; however, few have focused on the human in vivo response [33–35].

We were able to reduce the 48 h time point gene expression signature to just six genes (*CCL2*, *SEPT4*, *LAMP3*, *RTP4*, *MT1G* and *OAS3*). This enabled us to classify our samples into two distinct groups (symptomatic and asymptomatic) with 100 % accuracy providing a potentially convenient, cost effective and quantitative method for determining post-challenge outcome during vaccine and challenge trials. The reproducibility of this gene set has been demonstrated using samples from an independent vaccine study [17] with a high level of accuracy (100 % for asymptomatic and 89 % for symptomatic volunteers). To our knowledge, the only other transcriptomics analysis involving an H3N2 influenza challenge study was for 17 adults, described by Huang, Zaas, Woods and colleagues [10, 11, 17]. Woods et al. have also published data on H1N1 challenge [10]. They found the gene expression signatures induced by these two strains to be highly similar, with 88 % overlap between the most differentially expressed genes. Four of our six genes (*CCL2*, *LAMP3*, *RTP4* and *OAS3*) are represented in the list of the top 50 genes from this H1N1 study. Two studies in 2013 examined genome-wide gene expression in patients with influenza acquired in the community. While none of these six genes were amongst the best discriminators of respiratory syncytial virus (RSV) from influenza, four of the six genes (*OAS3*, *SEPT4*, *LAMP3* and *RPT4*) were represented in a gene list of 161 interferon-related differentially expressed genes [6]. In a separate study, Herberg et al. [5] reported genome-wide gene expression in 19 children with H1N1 infection. Of the 50 probes which were most significantly upregulated, four of our six genes were represented (*OAS3*, *LAMP3*, *SEPT4* and *MT1G*).

The *OAS3* gene product is an enzyme that functions to resist viral infection via the destruction of intracellular RNA [29]. *CCL2* encodes a chemokine that attracts monocytes. The clinical significance of CCL2/MCP1 in influenza disease has been suggested by studies that have associated elevated CCL2 levels with a poor prognosis in H1N1 infected Chinese patients [36] and a small interfering RNA (siRNA) screen showing effects on vaccinia virus infection [37]. While CCL2 attracts monocytes, receptor transporter protein 4 (RTP4) and the metallothionein isoform MT1G can be expressed by monocytes [38, 39]. RTP4 is known to be induced by type I interferons and have targeted anti-viral actions [40]. A high-throughput siRNA screen in primary lung cells showed that RPT4 was a key determinant of influenza virus replication [35]. *SEPT4* encodes a member of the septin family of nucleotide binding proteins, and Septin 5 was reported to be upregulated in chicken brain following H5N1 infection [41]. The lysosomal-associated membrane protein 3 (LAMP3) is expressed by activated DCs [42] and is significantly induced in human lung epithelial cells (A549 cells) following influenza infection in vitro [43]. Our network analysis and the published evidence described here links these genes to the immune and specifically anti-viral response but shows that they are not involved in the same biological pathways. By representing different biological pathways, these six genes may succinctly describe a complex pattern of gene expression.

The Huang dataset illustrates the variability that can be seen between individuals in response to influenza challenge. For the eight samples that we successfully predicted as being symptomatic using the panel of six genes, there is no one single time point at which all eight samples are predicted as symptomatic. This suggests that the window for symptomatic classification can vary from individual to individual over a number of hours. Further work may be required to optimise the panel of genes used dependent on the specific time window post-challenge used to measure gene expression.

Fewer vaccinated volunteers were expected to develop LCI and experience moderate/severe symptoms less frequently compared to unvaccinated volunteers. We did not detect a difference in the gene expression profile between vaccinated and unvaccinated individuals in this study. This is perhaps not surprising as initial samples were taken 30 days after vaccination, and previous studies [44] with viral-vectored vaccines have shown differential gene expression to be undetectable at 7 days post-vaccination. Further studies will be needed to confirm the preliminary evidence of vaccine efficacy, and these will be facilitated by the use of gene expression data to allow objective classification of outcomes.

This analysis gives further insight into host-pathogen interactions which could be beneficial for early detection of influenza. However, the major value is in the clinical trial setting. Current biological phenotyping methods often show inconsistency, but this work illustrates the use of a more quantitative method. The ability to better classify symptomatic individuals post influenza challenge will help accelerate future influenza vaccine efficacy studies.

## Electronic supplementary material

Below is the link to the electronic supplementary material.

ESM 1 (PDF 1255 kb)
