# Supplementary material for: Transcriptomic profiling facilitates classification of response to influenza challenge
Source: J Mol Med (Berl). 2014 Oct 28;93(1):105–14. doi: 10.1007/s00109-014-1212-8 (PMC4281383; doi:10.1007/s00109-014-1212-8)
Supplement: Supplementary file 1 — (PDF 1255 kb) [file 109_2014_1212_MOESM1_ESM.pdf]

Supplementary Materials for

Transcriptomic profiling facilitates classification of response to influenza challenge

Journal of Molecular Medicine

Emma E Davenport*, Richard D Antrobus*, Patrick J Lillie, Sarah Gilbert and Julian C Knight[#]

*These authors contributed equally
# Corresponding Author
Affiliation: Wellcome Trust Centre for Human Genetics, University of Oxford, Oxford, UK
Email: Julian@well.ox.ac.uk

The PDF file includes:

Table S1

Table S2

Table S3

Fig. S1

Fig. S2

Fig. S3

Table S1 HI titres following influenza challenge. HI titres are shown for day 26 after influenza challenge [1].

Volunteer ID	HI titre
A	40
B	<10
C	160
D	>640
E	>640
F	<10
G	226
H	80
I	20
J	320
K	<10
L	160
M	<10
N	80
O	320
P	40
Q	320
R	<10
S	20
T	160
U	20
V	80

Table S2 Differential gene expression in severe LCI. Numbers of differentially expressed probes and genes identified at different time points following challenge are shown for four individuals with moderate/severe LCI compared to the remaining individuals ($q < 0.05$ controlling for multiple testing using Benjamini and Hochberg's method).

Comparison (number of subjects)	Number of probes	Number of genes
12h post challenge moderate/severe LCI (4) vs not (18)	0	0
24h post challenge moderate/severe LCI (4) vs not (18)	0	0
48h post challenge moderate/severe LCI (4) vs not (18)	1103	952

Table S3 List of differentially expressed probes and genes. Differentially expressed probe IDs and gene names between the four moderate/severe LCI samples 48 hours post challenge and the remaining samples (n=1,103 probes q < 0.05).

Array address ID	Gene	log(fold change)	q value
1030333	*CCL2*	3.77	6.590E-16
1740360	*USP18*	4.33	9.290E-16
4670458	*SEPT4*	3.61	2.440E-15
7320546	*LAMP3*	4.96	5.280E-15
1980524	*GBP4*	2.94	1.910E-14
520059	*LAMP3*	3.22	3.930E-14
7380221	*SPATS2L*	3.40	2.830E-13
4150270	*ANKRD22*	3.57	8.240E-13
1990300	*SOCS1*	3.10	1.050E-12
1170300	*MT1G*	3.71	1.050E-12
130181	*ANKRD22*	4.03	6.220E-12
7160274	*TXNDC12*	-2.66	1.120E-11
6860482	*HERC6*	2.30	1.350E-11
630091	*NCOA7*	2.47	1.360E-11
6280543	*OASL*	3.12	2.900E-11
5900543	*SEPT4*	2.33	4.010E-11
3930681	*DHX58*	3.22	4.010E-11
1440615	*OTOF*	2.93	5.530E-11
2940059	*FTSJD2*	2.08	1.540E-10
4760703	*DDX58*	2.93	1.640E-10
4880010	*ZBP1*	2.76	1.910E-10
1450427	*RTP4*	2.61	2.140E-10
6200402	*MT1A*	2.94	2.960E-10
1570156	*OAS2*	2.46	3.160E-10
240722	*OAS2*	3.43	3.530E-10
5260070	*HES4*	3.81	4.040E-10
4780128	*ATF3*	2.80	4.040E-10
6110020	*USP41*	2.50	4.040E-10
6980133	*Hs.537991*	2.83	4.580E-10
3140041	*SP140*	2.13	6.340E-10
6580484	*MOV10*	2.37	7.110E-10
7320561	*OAS2*	2.82	7.110E-10
450615	*MT2A*	3.16	7.110E-10
6400176	*IRF7*	2.79	7.820E-10
2510523	*RBCK1*	1.87	8.530E-10
2070059	*PARP10*	2.21	9.510E-10
6180056	*TOR1B*	2.20	1.010E-09
7550242	*HESX1*	2.70	1.240E-09
2230204	*OAS2*	3.10	1.930E-09
3990010	*Hs.125087*	3.74	2.460E-09
3800398	*FBXO6*	2.67	2.620E-09

3180039	*RGL1*	1.94	3.290E-09
1440300	*SLC27A3*	1.79	3.600E-09
6620121	*CCL8*	3.95	4.430E-09
5700753	*CEACAM1*	2.63	4.490E-09
7330392	*TAP1*	1.62	5.020E-09
3060735	*IL1RN*	2.85	5.050E-09
2480450	*GALM*	2.77	5.210E-09
10360	*OAS3*	3.83	5.210E-09
630397	*KIAA0319L*	2.55	6.170E-09
6200672	*NUB1*	1.86	7.980E-09
1470382	*IRF7*	2.88	7.990E-09
1510196	*PHF11*	1.52	7.990E-09
4730059	*BATF2*	3.94	8.160E-09
4150014	*CEACAM1*	2.66	8.160E-09
380259	*INDO*	4.13	8.160E-09
6840035	*GBP1*	3.01	8.160E-09
2030309	*SERPING1*	4.55	8.160E-09
5340767	*CEACAM1*	2.85	9.050E-09
3940133	*FAM46A*	1.85	1.040E-08
580240	*TTC21A*	3.05	1.040E-08
2340072	*PARP12*	2.48	1.450E-08
150240	*TRIM78P*	1.75	1.800E-08
7560041	*AXUD1*	2.07	1.850E-08
1660010	*TRIM5*	2.24	1.940E-08
7400743	*KIAA1618*	2.14	1.990E-08
2360348	*CMPK2*	2.66	2.370E-08
2690435	*IFIT1*	2.51	2.840E-08
5220204	*C19orf66*	1.81	3.420E-08
5570711	*IDO1*	3.38	3.590E-08
7040035	*OAS1*	2.91	4.030E-08
1510364	*GBP5*	2.63	4.520E-08
620403	*LOC400759*	3.08	5.060E-08
360132	*LHFPL2*	1.95	5.770E-08
2510220	*IFI35*	2.82	5.860E-08
130519	*STAT2*	2.19	6.160E-08
6370768	*ETV7*	2.76	6.780E-08
2190397	*ZCCHC2*	2.43	6.780E-08
240053	*GCH1*	2.27	1.150E-07
4060050	*PNPT1*	1.83	1.210E-07
1090390	*OAS1*	3.14	1.240E-07
5670100	*TCN2*	2.09	1.430E-07
1030347	*IFITM4P*	2.44	1.490E-07
130048	*KIAA1958*	2.03	1.840E-07
2760537	*MTE*	2.58	1.940E-07
4890270	*LY6E*	2.58	2.070E-07
5960343	*PRIC285*	2.16	2.160E-07

450189	*XAF1*	2.55	2.270E-07
7610053	*DDX60*	2.21	2.510E-07
4540382	*ZC3HAV1*	1.84	2.690E-07
7650097	*OASL*	3.71	2.930E-07
7510537	*SCO2*	2.47	2.980E-07
4570441	*IFIH1*	2.59	3.020E-07
3710152	*TRIM5*	1.84	3.090E-07
10136	*PML*	2.47	3.450E-07
4050475	*KIAA0319L*	1.99	3.620E-07
5490470	*MX2*	2.06	4.260E-07
6220739	*GRAMD1B*	2.03	4.310E-07
5310100	*TDRD7*	1.76	4.590E-07
4150692	*PARP14*	2.32	4.770E-07
3420523	*RHBDF2*	1.46	5.170E-07
4900239	*CD274*	1.84	5.380E-07
5550397	*APOL6*	1.94	5.770E-07
4860224	*WARS*	2.66	6.360E-07
6520523	*IL27*	1.83	6.550E-07
150400	*LBA1*	1.90	6.650E-07
4900435	*TNFSF13B*	2.06	8.490E-07
4850228	*LOC647135*	2.13	8.540E-07
380050	*RHBDF2*	1.57	8.540E-07
2630195	*VAMP5*	2.25	8.740E-07
1030100	*Hs.386275*	2.67	9.170E-07
6270553	*CXCL10*	3.74	9.400E-07
3800487	*ZNFX1*	1.75	1.200E-06
870202	*TNFSF10*	2.31	1.270E-06
3310450	*LOC648366*	1.88	1.350E-06
2070170	*UBE2L6*	1.97	1.430E-06
5820753	*RBCK1*	1.82	1.580E-06
6860162	*LOC441019*	2.29	1.690E-06
4200240	*SP100*	1.97	1.710E-06
2340332	*SRGAP2L*	1.84	1.900E-06
5890241	*ZFYVE26*	1.40	1.980E-06
3170136	*SAMD9L*	2.35	2.580E-06
4780044	*LOC389386*	2.72	2.630E-06
5910064	*TRIM5*	1.84	2.630E-06
3710068	*WARS*	2.84	2.650E-06
5090215	*IFI6*	2.03	2.750E-06
1990487	*XRN1*	1.53	3.080E-06
6100521	*PHF11*	1.39	3.110E-06
6840020	*TNFSF13B*	1.87	3.300E-06
1570484	*ATF5*	1.78	3.490E-06
2470601	*IL1RN*	3.19	3.640E-06
1070767	*OAS1*	3.21	4.260E-06
4060047	*TNFAIP6*	2.17	4.260E-06

6520110	*PHF11*	1.55	4.300E-06
6290743	*APOBEC3F*	1.70	4.610E-06
5560270	*CARD17*	1.61	4.640E-06
3890609	*PLSCR1*	2.58	4.940E-06
5220497	*ACOT9*	1.49	5.000E-06
5720131	*C9orf91*	2.05	5.840E-06
4560541	*MLKL*	1.29	5.950E-06
3710243	*ZC3HAV1*	2.14	5.950E-06
6040653	*TRIM6*	2.12	7.060E-06
990768	*OAS3*	3.71	7.110E-06
3930377	*TRIM38*	1.43	7.520E-06
5310427	*PATL1*	1.28	7.550E-06
4290730	*LGALS3BP*	1.98	7.920E-06
3870594	*IFI16*	1.53	8.000E-06
6560451	*HNRPLL*	1.82	8.120E-06
6220332	*SLC6A12*	1.59	9.160E-06
4290368	*PSTPIP2*	1.59	1.010E-05
7000368	*UBE2L6*	2.09	1.050E-05
2490372	*PLAC8*	1.35	1.150E-05
1230091	*PML*	1.54	1.200E-05
870193	*SP140*	2.07	1.200E-05
1260270	*AIM2*	2.15	1.200E-05
4570164	*LOC389386*	2.39	1.360E-05
1990397	*SP110*	1.74	1.380E-05
1570129	*TRAFD1*	1.85	1.400E-05
6330132	*ISG20*	1.72	1.400E-05
4060632	*C17orf87*	1.42	1.400E-05
4890598	*BRSK1*	1.69	1.750E-05
1820750	*STAT1*	2.09	1.750E-05
6650541	*SP100*	1.61	1.750E-05
5570398	*FCGR1C*	2.80	1.930E-05
7160593	*PPM1K*	1.43	1.930E-05
6770707	*SQRDL*	1.68	2.000E-05
770253	*KIAA1618*	1.99	2.150E-05
2900450	*ADAR*	1.23	2.380E-05
6220152	*ANKFY1*	1.83	2.450E-05
2140242	*TNFAIP6*	2.58	2.500E-05
2100196	*ISG15*	3.67	2.600E-05
520408	*IFIT3*	3.06	2.890E-05
6060142	*AFF1*	1.48	2.890E-05
4920110	*GADD45B*	1.44	2.980E-05
4850494	*LOC643384*	1.60	2.980E-05
5700725	*EPSTI1*	2.91	2.990E-05
5960747	*TRIM22*	1.95	3.020E-05
20441	*Hs.254477*	1.86	3.220E-05
5220189	*PIK3AP1*	1.45	3.220E-05

130753	*AIM2*	1.87	3.270E-05
1820114	*LILRB4*	1.53	3.270E-05
450300	*ANKFY1*	1.39	3.300E-05
5490433	*ADAR*	1.44	3.460E-05
3780047	*GBP6*	1.86	3.730E-05
3850059	*LMO2*	1.53	3.730E-05
3290292	*LAP3*	2.70	3.820E-05
5870184	*SP110*	1.67	3.910E-05
2120079	*EIF2AK2*	2.09	3.990E-05
150647	*NTNG2*	1.88	4.060E-05
2360392	*SLC25A28*	1.22	4.120E-05
3930601	*PARP10*	1.99	4.280E-05
6620209	*FCGR1B*	2.52	4.370E-05
3170273	*FER1L3*	2.05	4.440E-05
450468	*MRPL44*	1.08	4.730E-05
2230367	*CERK*	-0.92	4.910E-05
1580400	*TTC26*	1.71	5.090E-05
5360156	*IFITM1*	1.33	5.100E-05
3180681	*NCF1C*	1.68	5.610E-05
7650026	*MUC1*	1.32	5.720E-05
5890678	*C17orf87*	1.48	5.860E-05
3400270	*RNF213*	2.08	6.180E-05
2070646	*GPR84*	1.36	6.180E-05
2680523	*SAMD4A*	1.56	6.190E-05
2710328	*MLKL*	1.57	6.280E-05
1090132	*PARP10*	2.11	6.380E-05
3420632	*PSMB8*	1.17	6.940E-05
460243	*UNC93B1*	1.54	6.940E-05
7550022	*STXBP5*	-1.58	6.970E-05
7610309	*Hs.20255*	1.46	7.560E-05
1010246	*IFI6*	3.11	7.840E-05
6510735	*DDX60L*	2.01	7.980E-05
4860220	*SRGAP2*	1.47	8.480E-05
4610608	*LOC731049*	1.43	8.550E-05
2190148	*GBP1*	2.35	8.550E-05
1980309	*IL8*	-2.96	8.590E-05
770538	*LYSMD2*	1.29	9.460E-05
5050735	*TMEM62*	1.41	9.750E-05
3360343	*RSAD2*	4.16	9.850E-05
4040386	*APOL1*	1.89	1.006E-04
7610440	*XAF1*	2.33	1.014E-04
5290176	*SIGLEC1*	1.43	1.016E-04
1340491	*SP110*	1.44	1.016E-04
4810435	*SCARB2*	1.40	1.025E-04
3450180	*OAS1*	2.84	1.026E-04
5900524	*LACTB*	1.51	1.027E-04

7560296	*RBM43*	1.61	1.027E-04
6510170	*IFIT3*	3.42	1.052E-04
6400681	*SP140*	1.90	1.052E-04
3940520	*NT5C3*	1.79	1.070E-04
7610050	*ABCC5*	-1.30	1.161E-04
6100022	*HIST2H2AC*	1.58	1.189E-04
4390450	*SGK*	-1.45	1.218E-04
2570079	*STAT1*	1.57	1.310E-04
6760286	*Hs.72010*	1.47	1.352E-04
1430039	*KIAA0319L*	1.70	1.383E-04
7210326	*PSMB8*	1.10	1.383E-04
3390121	*CASP5*	2.53	1.386E-04
4670592	*MYOF*	1.88	1.404E-04
3170064	*MICB*	1.33	1.421E-04
60470	*STX11*	1.89	1.530E-04
1010360	*GCH1*	2.07	1.654E-04
2490450	*LOC91561*	-1.77	1.661E-04
6840753	*SPTLC2*	1.30	1.661E-04
2850202	*UBA7*	1.07	1.661E-04
4900630	*KIAA0319L*	1.99	1.661E-04
6350634	*DYNLT1*	1.30	1.661E-04
4850592	*P2RY14*	1.90	1.791E-04
6980113	*PHACTR2*	1.38	1.791E-04
5690671	*GOLGA8B*	-1.53	1.795E-04
3840593	*SP110*	1.43	1.852E-04
6590646	*FAM26F*	2.15	1.852E-04
5420538	*TP53INP1*	-1.25	1.970E-04
3290551	*EIF4G3*	1.38	2.068E-04
5820424	*LOC100129681*	1.91	2.185E-04
1440736	*LDLR*	1.34	2.185E-04
160731	*SHISA5*	1.36	2.310E-04
2630224	*LOC93622*	-1.47	2.310E-04
2510239	*TRIM56*	1.35	2.341E-04
4830113	*PLAC8*	1.15	2.423E-04
6270554	*LGALS8*	1.25	2.647E-04
6860180	*LOC728216*	2.37	2.668E-04
6380594	*LOC641922*	1.57	2.688E-04
4890327	*IFIT5*	1.95	2.688E-04
1410209	*SGK1*	-1.67	2.699E-04
4610102	*TMEM123*	1.30	2.818E-04
1010253	*NT5C2*	1.09	2.959E-04
3390017	*CNDP2*	1.18	2.967E-04
1820592	*HIST2H2AA3*	1.69	2.967E-04
4900674	*BST2*	2.02	3.091E-04
840360	*UBQLNL*	1.71	3.199E-04
1090367	*BIVM*	-1.22	3.320E-04

3290008	*ERN1*	-1.11	3.414E-04
5870192	*SKAP1*	-1.27	3.416E-04
2190221	*EIF3L*	-0.99	3.416E-04
6380086	*GORASP1*	1.41	3.459E-04
990072	*SRGAP2*	1.35	3.459E-04
4670414	*TMEM140*	1.71	3.485E-04
540020	*LGALS8*	1.15	3.490E-04
1300246	*DISC1*	2.30	3.530E-04
1580750	*LOC100130828*	1.89	3.891E-04
840431	*LGALS9*	1.82	3.891E-04
4810187	*STAT1*	1.45	3.948E-04
2070288	*MT1E*	1.71	4.027E-04
6840767	*FRMD3*	2.34	4.035E-04
2060440	*MAFB*	1.69	4.134E-04
2710709	*FCGR1B*	2.50	4.178E-04
4780427	*KIAA0082*	1.50	4.235E-04
4780612	*UNC93B1*	1.61	4.333E-04
1190739	*ITPRIPL2*	1.49	4.333E-04
3850066	*KIAA0226*	1.27	4.369E-04
6450424	*NME3*	-0.88	4.618E-04
6510446	*PRKD2*	1.46	4.669E-04
1410168	*ELF4*	1.06	4.669E-04
3130291	*C12orf57*	-1.20	4.839E-04
7160592	*ACOT9*	1.72	4.857E-04
3140707	*PARP9*	1.73	4.857E-04
7510647	*CACNA1E*	1.60	4.960E-04
7000228	*LOC400455*	-0.98	5.085E-04
6020367	*LOC100129334*	1.11	5.092E-04
5490202	*LOC646688*	-1.20	5.297E-04
5700735	*PARP9*	1.65	5.505E-04
1850220	*DAPP1*	1.39	5.527E-04
7560471	*MOBKL2C*	1.39	5.587E-04
3940477	*TAP2*	1.40	5.587E-04
3060692	*CERK*	-0.93	5.587E-04
5810288	*LOC643384*	1.43	5.598E-04
5390161	*DUSP5*	1.08	5.612E-04
2030324	*SGK1*	-1.46	5.670E-04
4560576	*MYD88*	1.08	5.819E-04
3870338	*IFI44L*	3.77	5.927E-04
2450301	*LOC730029*	-1.08	5.976E-04
5690181	*KIAA1712*	-1.38	6.049E-04
520086	*FCGR1A*	2.71	6.294E-04
70601	*CUL1*	0.94	6.566E-04
6250064	*IRF1*	1.03	6.580E-04
2760500	*CD38*	1.60	6.930E-04
7650669	*TBC1D4*	-1.32	7.138E-04

7400102	*ASPHD2*	1.20	7.183E-04
6180497	*LOC400304*	-1.84	7.378E-04
5390730	*PIK3IP1*	-1.40	7.484E-04
6110736	*IRS2*	-1.32	7.528E-04
3190112	*SERPINB1*	1.13	7.714E-04
2230678	*ACACB*	-1.41	8.318E-04
3610333	*LOC441013*	-1.43	8.741E-04
4120754	*DCUN1D4*	-1.06	8.853E-04
2750326	*OR52K2*	1.58	8.878E-04
4120673	*TRIM14*	1.65	9.189E-04
6560291	*FAR2*	1.29	9.189E-04
830609	*RPL5*	-1.58	9.314E-04
6480615	*IER2*	1.15	9.358E-04
4560717	*MDK*	1.23	9.483E-04
5720482	*HERC5*	2.97	9.716E-04
3130307	*TYMP*	1.88	9.978E-04
4210619	*CD2*	-1.04	1.040E-03
4010020	*ELF1*	1.00	1.043E-03
2350189	*GBP3*	1.85	1.044E-03
7320176	*CCR1*	1.61	1.045E-03
3420739	*SNTB1*	1.15	1.051E-03
6960168	*ATP8B2*	-1.08	1.071E-03
6560075	*TRIM21*	1.40	1.091E-03
3180026	*PSMB8*	1.23	1.105E-03
4610241	*P2RY14*	1.71	1.160E-03
4640411	*KCND1*	1.14	1.189E-03
1190026	*APOBEC3G*	1.21	1.273E-03
7330689	*LOC728672*	-1.09	1.278E-03
1820681	*DPEP2*	-1.07	1.278E-03
5570039	*LOC728744*	1.85	1.278E-03
6860246	*GRAMD1C*	-1.75	1.278E-03
20246	*LILRB1*	1.56	1.288E-03
990138	*PRKD2*	1.34	1.312E-03
2810521	*LOC647276*	-0.97	1.343E-03
1510154	*TCFL5*	-1.30	1.345E-03
840433	*RSPH9*	1.17	1.368E-03
3180131	*LOC389672*	-1.38	1.384E-03
2940068	*GPBAR1*	1.40	1.384E-03
4290148	*HIST2H2AA4*	1.39	1.384E-03
1190040	*GIMAP8*	1.37	1.384E-03
3450041	*RNF114*	0.86	1.385E-03
2490315	*Hs.306876*	1.11	1.390E-03
5810022	*MLKL*	1.30	1.439E-03
7100471	*TSTD1*	-1.26	1.440E-03
2120465	*RNF114*	0.78	1.459E-03
610451	*HIST2H2AA3*	1.38	1.463E-03

770088	*DTX3L*	1.65	1.463E-03
2690324	*ITPRIP*	1.05	1.463E-03
1850482	*TNK2*	1.01	1.469E-03
4150110	*KIAA0114*	-1.00	1.469E-03
3400632	*GRIN3A*	1.17	1.473E-03
1450246	*IRF2*	1.24	1.487E-03
3420593	*LMNB1*	1.47	1.561E-03
3940484	*MEF2D*	-1.00	1.594E-03
2760121	*ADHFE1*	-1.20	1.600E-03
240064	*ETV6*	0.97	1.625E-03
70730	*GAS6*	1.07	1.625E-03
6940605	*Hs.489254*	1.17	1.658E-03
1410537	*RPSA*	-1.10	1.669E-03
1510608	*Hs.371609*	-1.04	1.676E-03
4540072	*C16orf75*	1.27	1.681E-03
6020463	*TRIM26*	1.12	1.691E-03
2230725	*LOC728031*	-1.13	1.761E-03
2000445	*PSMB10*	0.89	1.779E-03
1570358	*APOL2*	1.35	1.839E-03
7160468	*DHRS9*	1.49	1.866E-03
5560079	*GPBAR1*	1.36	1.868E-03
3290687	*ZNF84*	-1.28	1.868E-03
7150082	*LOC285296*	1.44	1.878E-03
4250451	*SEC24D*	1.23	1.878E-03
7100300	*ZNF200*	1.17	1.893E-03
3990598	*DRAP1*	1.37	1.911E-03
6650324	*PLEKHA1*	-1.28	1.917E-03
4250445	*RPL4*	-1.16	1.941E-03
1710242	*C1orf59*	-1.14	1.941E-03
450414	*PPM1H*	-1.26	1.949E-03
6450692	*PSMB9*	1.21	1.981E-03
3850762	*LOC649076*	-0.87	2.037E-03
450010	*LOC390578*	-1.34	2.037E-03
940504	*RUNX2*	-1.35	2.082E-03
20451	*MEFV*	1.32	2.086E-03
2000424	*Hs.569566*	1.35	2.101E-03
1570300	*SPATA13*	1.33	2.110E-03
730010	*ADPRHL2*	1.02	2.110E-03
610148	*GSDMD*	1.07	2.110E-03
3890382	*ST3GAL5*	0.95	2.164E-03
650441	*GTPBP2*	1.39	2.166E-03
2030170	*CARD16*	1.44	2.174E-03
1500725	*CD1C*	-1.30	2.181E-03
1990438	*PANK2*	1.03	2.182E-03
3850246	*HOPX*	-1.86	2.184E-03
2000768	*FAR2*	1.35	2.184E-03

5390494	*EIF4B*	-1.02	2.193E-03
6330315	*FLJ39639*	-0.90	2.279E-03
110095	*GMCL1*	-0.77	2.303E-03
7200753	*TLR7*	1.51	2.309E-03
3310376	*TIMM10*	2.80	2.315E-03
4290382	*TMEM131*	0.79	2.318E-03
2350504	*ECGF1*	1.20	2.319E-03
6020092	*LOC100133045*	1.20	2.319E-03
6180050	*STK24*	0.74	2.407E-03
5310411	*H2AFJ*	1.25	2.472E-03
7550358	*NELL2*	-1.59	2.478E-03
7150152	*PTPLAD1*	-0.99	2.612E-03
7210093	*PGM2L1*	-1.11	2.664E-03
5550768	*Hs.294603*	-1.01	2.692E-03
6040647	*HSH2D*	1.32	2.692E-03
5810600	*MAP3K5*	0.93	2.692E-03
4250446	*LOC388707*	-1.07	2.762E-03
7560632	*ITK*	-1.13	2.780E-03
7400630	*BCKDHB*	0.97	2.893E-03
3370086	*P2RX7*	1.38	2.955E-03
160019	*SORT1*	1.37	2.955E-03
7000398	*NT5C3*	1.65	2.980E-03
270224	*MOAP1*	-1.03	3.049E-03
540075	*NPC2*	0.94	3.060E-03
6980753	*NADK*	1.19	3.089E-03
2360647	*TFEC*	1.39	3.144E-03
730138	*CXorf21*	1.47	3.160E-03
4920228	*FFAR2*	1.39	3.211E-03
5910019	*C1QB*	1.45	3.293E-03
7210717	*FGD2*	1.00	3.301E-03
3890523	*IL7R*	-1.43	3.333E-03
2370072	*PRR11*	1.34	3.362E-03
1570746	*LOC651816*	1.26	3.400E-03
6520026	*NUCB1*	1.00	3.501E-03
2970347	*ITPRIP*	1.09	3.526E-03
4150520	*RIPK2*	0.98	3.593E-03
3990672	*LOC440595*	-0.98	3.989E-03
6770689	*LOC653162*	-0.90	3.989E-03
5270681	*TMEM110*	1.20	3.989E-03
5900497	*PLXDC2*	1.02	4.062E-03
6510707	*FER1L3*	1.30	4.101E-03
4200195	*ZNF684*	1.48	4.134E-03
3610386	*CPEB3*	1.00	4.278E-03
2600747	*IFIT2*	1.93	4.278E-03
1500280	*IFIT3*	2.96	4.305E-03
2970019	*HIST1H4H*	1.51	4.332E-03

6620750	*ZC3HAV1*	1.04	4.390E-03
3830095	*DFFB*	-0.89	4.390E-03
3130309	*LOC100132804*	-1.52	4.436E-03
6550315	*LYRM1*	0.79	4.449E-03
3990433	*ADAP2*	1.15	4.603E-03
1300349	*SOCS4*	1.09	4.658E-03
1570327	*BRCA2*	1.13	4.676E-03
3890255	*TOP2B*	-1.10	4.792E-03
6290349	*LOC389223*	-0.87	4.803E-03
4230102	*SOCS3*	1.09	4.828E-03
3360504	*EEF1A1*	-1.20	4.844E-03
1990403	*LOC730704*	1.19	4.861E-03
5220538	*WDFY3*	1.19	4.861E-03
3370066	*APOL2*	1.31	4.861E-03
1190138	*TXK*	-1.23	4.861E-03
6450129	*ITM2A*	-1.51	4.861E-03
3930368	*CARD17*	1.19	4.861E-03
1410750	*EEF1AL7*	-0.93	4.950E-03
6060356	*RPL13A*	-1.25	4.950E-03
3930349	*SDHAF1*	-0.78	5.108E-03
580463	*KIAA0895L*	1.35	5.147E-03
3930187	*LOC729196*	1.03	5.171E-03
2900292	*RANGAP1*	1.02	5.186E-03
7560593	*OSM*	1.36	5.337E-03
630315	*DHRS9*	1.36	5.420E-03
2340400	*LOC284393*	-0.69	5.472E-03
650750	*RHBDF2*	0.90	5.472E-03
7150678	*RAB8A*	0.88	5.472E-03
7560288	*DCUN1D3*	0.95	5.506E-03
5700020	*BTBD15*	-1.00	5.679E-03
6370435	*ETS1*	-1.08	5.749E-03
7000328	*LOC100129550*	-0.99	5.757E-03
3440670	*LOC402251*	-1.09	5.775E-03
4180100	*APPL2*	-0.96	5.778E-03
10008	*RERE*	1.03	5.784E-03
7380243	*LOC728772*	0.86	5.784E-03
6180095	*LOC730029*	-1.27	5.874E-03
2340035	*LOC644464*	-0.84	5.939E-03
6580553	*ABCC5*	-1.15	5.962E-03
1770243	*IGF2BP3*	1.44	6.056E-03
1500747	*CBX7*	-0.91	6.076E-03
6840577	*KPNB1*	0.85	6.164E-03
2570300	*IFI44*	2.67	6.183E-03
460750	*PDCL3*	-1.06	6.197E-03
620609	*LOC387867*	-0.96	6.197E-03
5550474	*RAB24*	1.15	6.319E-03

6840324	*RPL13L*	-0.94	6.426E-03
3370136	*ACAD11*	-1.03	6.435E-03
830022	*SLC12A2*	-1.22	6.445E-03
6840184	*GRN*	1.05	6.579E-03
2710152	*ODF3B*	1.09	6.586E-03
6560554	*LOC100132516*	1.19	6.831E-03
6660630	*TP53INP1*	-1.07	6.921E-03
6960017	*SLC6A13*	1.33	6.989E-03
6900356	*ZNF529*	-0.89	6.998E-03
7650128	*ECGF1*	1.68	6.998E-03
2320689	*LOC653610*	1.29	7.013E-03
2370192	*LOC441506*	-1.08	7.082E-03
4570255	*LEF1*	-1.22	7.082E-03
2190612	*TMEM229B*	1.17	7.089E-03
1430711	*KLF12*	-1.68	7.149E-03
1230347	*RERE*	1.01	7.149E-03
7320370	*STAT4*	-1.10	7.242E-03
6980500	*Hs.520349*	-1.17	7.342E-03
460373	*LACTB*	1.02	7.414E-03
6650242	*IFITM3*	3.02	7.418E-03
3520020	*CYFIP2*	-1.04	7.452E-03
6280370	*LOC646688*	-1.06	7.506E-03
2650400	*LOC648249*	-1.05	7.506E-03
4570475	*LOC649049*	-1.17	7.550E-03
4640576	*PRKCQ*	-0.99	7.580E-03
2650156	*TAP2*	1.42	7.621E-03
5260138	*NSF*	0.73	7.621E-03
4880433	*IDH2*	1.10	7.635E-03
2810400	*KLHL3*	-1.31	7.830E-03
2030008	*LOC440595*	-0.97	7.900E-03
50706	*CD40LG*	-1.19	7.915E-03
5130471	*PDE3B*	-1.32	7.967E-03
5340414	*LGTN*	-0.92	8.056E-03
6380148	*FAM104B*	0.83	8.153E-03
4280632	*GAS6*	1.14	8.181E-03
540747	*Hs.276860*	-0.85	8.181E-03
3870390	*CACNA1A*	1.08	8.227E-03
1300441	*ZZZ3*	-1.19	8.277E-03
6130040	*LOC388282*	1.00	8.277E-03
2060291	*STOM*	1.20	8.291E-03
160754	*EDG1*	-1.05	8.350E-03
6580044	*PTPN22*	-1.46	8.391E-03
4860255	*ASPRV1*	1.55	8.444E-03
2810471	*PTGDR*	-1.63	8.469E-03
20521	*MASTL*	1.12	8.469E-03
4040187	*C6orf190*	-1.38	8.493E-03

6620563	*LOC644604*	-0.78	8.493E-03
7150017	*C6orf48*	-0.98	8.511E-03
6040195	*LOC647856*	-1.06	8.534E-03
670594	*C2*	1.19	8.585E-03
3840484	*LOC652287*	1.06	8.880E-03
4250750	*LOC388524*	-0.75	8.901E-03
2000022	*IRF9*	0.94	8.913E-03
5570427	*GLS*	-1.25	8.950E-03
360653	*LOC647276*	-0.77	8.971E-03
4070132	*PPAT*	-1.00	9.033E-03
5420564	*NFIL3*	1.24	9.112E-03
1240504	*JMJD1A*	-0.84	9.112E-03
1030296	*BCL11B*	-1.05	9.112E-03
6590445	*RIN2*	1.65	9.238E-03
4210703	*RPL15*	-0.99	9.375E-03
1580373	*NAPA*	1.28	9.376E-03
2810632	*PTK2B*	1.02	9.434E-03
4290072	*SERTAD1*	0.84	9.443E-03
7000274	*LOC644039*	-0.73	9.443E-03
830750	*NCF1B*	1.45	9.443E-03
630619	*HPSE*	1.39	9.443E-03
5080372	*LOC646672*	-0.89	9.443E-03
6400603	*PVRIG*	-0.87	9.443E-03
6580164	*PHF21A*	0.79	9.562E-03
4180142	*KIAA1370*	-1.32	9.584E-03
6180253	*CDC42EP2*	1.27	9.609E-03
5490431	*SAT1*	0.88	9.648E-03
1340608	*LOC728126*	-0.98	9.648E-03
4250343	*PPP1R2*	-1.06	9.648E-03
6580332	*RNF103*	-1.03	9.680E-03
5910546	*MGC4677*	1.18	9.844E-03
1820242	*C10orf21*	1.23	1.017E-02
4040719	*DNAJC25*	-1.42	1.020E-02
160403	*CARS2*	0.75	1.020E-02
2230730	*SRBD1*	0.98	1.020E-02
6370739	*MACF1*	-1.01	1.027E-02
6420524	*LOC643873*	-1.00	1.037E-02
2750187	*NEXN*	1.30	1.037E-02
1430278	*CTSK*	-1.09	1.041E-02
5870451	*LOC100130624*	-0.88	1.044E-02
3170689	*NKTR*	-1.03	1.044E-02
4760091	*CEP78*	-1.18	1.049E-02
3390687	*MEFV*	1.43	1.056E-02
7380689	*RPL22*	-0.97	1.056E-02
1170671	*CD3D*	-1.47	1.056E-02
1780324	*LOC646784*	-1.57	1.056E-02

6560373	*RAB24*	0.98	1.058E-02
2190541	*GATS*	-0.92	1.061E-02
1230196	*AHCTF1*	-1.39	1.061E-02
6110731	*ITPK1*	1.21	1.061E-02
4670603	*ELMO2*	0.79	1.061E-02
1570553	*IL8*	-1.58	1.062E-02
7610131	*EPAS1*	1.16	1.066E-02
6200255	*PECR*	-0.98	1.072E-02
3800110	*ORC5L*	-0.90	1.081E-02
770520	*TAF4*	-0.82	1.082E-02
2230767	*LOC387825*	-1.30	1.090E-02
1010450	*RNY1*	0.95	1.091E-02
20692	*HINT3*	0.94	1.106E-02
5820646	*CALCOCO2*	0.86	1.114E-02
2760079	*FOXC1*	1.13	1.116E-02
4810768	*S1PR1*	-1.12	1.121E-02
1230209	*LOC100128410*	-1.15	1.124E-02
1850463	*LOC732371*	0.97	1.141E-02
3450280	*PLEKHO1*	0.78	1.150E-02
4890433	*LOC729679*	-0.92	1.153E-02
520603	*LOC401537*	-1.08	1.153E-02
1240553	*SESN1*	-1.54	1.161E-02
1690044	*CA5B*	-0.94	1.168E-02
3710187	*DISC1*	1.15	1.222E-02
4050435	*LOC645715*	-0.89	1.222E-02
2650082	*TAPBP*	0.98	1.224E-02
4200463	*Hs.126768*	-1.18	1.227E-02
6110392	*GNS*	0.84	1.230E-02
3390075	*PTPN4*	-1.19	1.231E-02
1050184	*NUB1*	0.97	1.236E-02
270026	*RASSF1*	-1.13	1.236E-02
3440360	*ZNF438*	1.01	1.236E-02
6960554	*LOC646793*	0.86	1.236E-02
2760239	*RASGRP1*	-1.22	1.240E-02
3850433	*HSPA1B*	1.23	1.242E-02
6650341	*FCGR2B*	1.14	1.245E-02
770209	*LOC729686*	-1.13	1.245E-02
4730341	*KIF27*	-1.03	1.246E-02
3710397	*EFNA1*	-1.28	1.247E-02
2000300	*IFI30*	1.21	1.254E-02
4610273	*CLYBL*	-0.86	1.266E-02
940148	*ABTB2*	1.07	1.266E-02
3850121	*EEF1A1*	-0.80	1.282E-02
1240142	*SAMD9*	1.44	1.288E-02
4050491	*TCEA3*	-1.48	1.288E-02
7040647	*SLC26A11*	-0.97	1.288E-02

2750750	*UBE2G2*	-0.89	1.299E-02
2190020	*GNG2*	-1.37	1.304E-02
2640397	*CXorf21*	1.06	1.304E-02
4830709	*Hs.224794*	1.05	1.304E-02
3520170	*SNORD87*	-1.08	1.304E-02
2810364	*KLHDC2*	-1.12	1.304E-02
10612	*RBM17*	-1.05	1.304E-02
150047	*PSME1*	0.93	1.311E-02
10504	*FGFBP2*	-2.05	1.341E-02
2850435	*SIAH1*	-0.94	1.342E-02
4150201	*BCL2*	-0.92	1.342E-02
60524	*DENND1A*	1.37	1.349E-02
2370114	*LOC441013*	-0.82	1.349E-02
4260093	*ZCCHC11*	-0.99	1.349E-02
580433	*DENND1A*	1.29	1.359E-02
610379	*APOBEC3F*	1.11	1.371E-02
1740382	*GCH1*	1.13	1.397E-02
2340750	*ESCO1*	-1.04	1.397E-02
3440095	*PSG3*	1.22	1.397E-02
130414	*LOC730167*	1.35	1.397E-02
6560220	*PTPN22*	-1.21	1.397E-02
4780133	*GBA*	0.90	1.405E-02
20452	*TYMP*	1.60	1.405E-02
1690435	*LOC441506*	-1.13	1.406E-02
6220671	*PLAUR*	1.29	1.408E-02
6940358	*SELL*	1.20	1.411E-02
4290575	*C7orf23*	-0.92	1.427E-02
5270520	*FAIM3*	-0.99	1.427E-02
3290731	*PRKCH*	-0.91	1.438E-02
510739	*INPP5A*	-0.92	1.440E-02
2060341	*AKAP13*	0.87	1.440E-02
4230672	*LOC653496*	0.81	1.457E-02
1770592	*RPGR*	-1.48	1.468E-02
6580292	*LOC729342*	-1.12	1.477E-02
2600470	*WDR60*	-1.07	1.477E-02
1940180	*PSMB9*	1.53	1.479E-02
4250056	*TRIM14*	1.20	1.487E-02
3440446	*C9orf156*	-0.79	1.493E-02
3120671	*RPA1*	-0.83	1.493E-02
5340435	*STOML1*	1.11	1.509E-02
1190626	*ZMYND11*	-1.10	1.540E-02
4120369	*APOBEC3G*	1.13	1.544E-02
5890193	*MS4A4A*	1.44	1.548E-02
7160292	*ST3GAL2*	0.98	1.561E-02
3420259	*MIR21*	1.03	1.562E-02
1340364	*P2RY14*	1.26	1.562E-02

3130474	*PANK2*	0.95	1.562E-02
7040392	*EXOC3L*	1.16	1.563E-02
3460167	*NAP1L1*	-1.02	1.578E-02
510224	*LPIN2*	0.89	1.578E-02
5050603	*TBC1D10C*	-0.71	1.579E-02
1660681	*CCDC102A*	-1.14	1.591E-02
5910465	*SH2D1A*	-1.40	1.591E-02
1770367	*LOC652699*	1.17	1.591E-02
5090632	*LGALS9*	1.45	1.599E-02
3840519	*DPEP3*	-1.31	1.622E-02
1690066	*MX1*	2.06	1.623E-02
3310228	*C11orf67*	1.03	1.623E-02
510100	*CBY1*	-1.02	1.623E-02
2370414	*C16orf42*	-0.82	1.624E-02
6370202	*RBBP4*	-0.91	1.626E-02
5820482	*LOC153684*	1.04	1.655E-02
1010672	*NR3C2*	-1.07	1.686E-02
7560341	*KREMEN2*	1.14	1.691E-02
4670170	*LOC730754*	-0.99	1.703E-02
6760021	*SNHG7*	-1.16	1.706E-02
7000202	*P4HTM*	-0.77	1.708E-02
4390612	*CCDC14*	-1.25	1.709E-02
4390349	*ZNF256*	-1.25	1.712E-02
1240097	*BAZ1A*	0.99	1.712E-02
5310747	*PSIP1*	-1.39	1.717E-02
2480717	*FCGR2B*	1.05	1.730E-02
5900373	*LOC389141*	-0.90	1.732E-02
70753	*C9orf123*	-1.20	1.744E-02
6200743	*CNPY4*	-1.04	1.744E-02
4230678	*HIST2H2BE*	1.13	1.744E-02
2480451	*IFITM4P*	1.01	1.749E-02
4490014	*TTC13*	-1.20	1.749E-02
780259	*ZNHIT6*	-0.99	1.755E-02
6280717	*LOC651198*	-0.75	1.762E-02
580470	*MASTL*	1.18	1.762E-02
6420309	*FAM134B*	-1.18	1.784E-02
450445	*NSUN5*	-0.88	1.785E-02
4040504	*POLR1E*	-1.05	1.785E-02
10326	*KIF22*	-0.77	1.787E-02
4150615	*LOC100132673*	-0.91	1.789E-02
4540327	*RRBP1*	1.00	1.792E-02
1070451	*ANXA5*	0.92	1.795E-02
3120370	*CD68*	0.90	1.797E-02
4280273	*GM2A*	1.33	1.798E-02
4280603	*SLBP*	-0.83	1.812E-02
3120279	*PRKAG2*	1.01	1.812E-02

4560047	*CD74*	1.45	1.812E-02
3840554	*SPOCK2*	-0.83	1.816E-02
3710647	*MXD4*	-0.99	1.816E-02
1400593	*SIGLEC14*	1.41	1.820E-02
5390707	*DIDO1*	-0.81	1.820E-02
3420368	*LOC644464*	-0.82	1.830E-02
5910300	*RAB24*	0.96	1.831E-02
4900747	*C11orf75*	1.26	1.831E-02
6560156	*DUSP3*	0.84	1.834E-02
7550008	*RNF216*	-0.75	1.837E-02
1740280	*NIT1*	0.82	1.855E-02
6480059	*ACTA2*	1.57	1.856E-02
4010243	*NAE1*	-1.27	1.856E-02
7200435	*ASCL2*	1.20	1.866E-02
6130075	*LOC391769*	0.95	1.866E-02
4210630	*ARHGAP23*	1.10	1.870E-02
870767	*TMEM194A*	-1.25	1.878E-02
580102	*Hs.554324*	-1.02	1.902E-02
6180592	*Hs.143408*	-1.08	1.902E-02
840685	*IL1B*	1.30	1.902E-02
110338	*XRN1*	1.18	1.902E-02
2070044	*CLYBL*	-1.25	1.909E-02
510196	*SPATA12*	0.90	1.909E-02
4480520	*HLTF*	-1.24	1.928E-02
780324	*ZNF337*	-1.09	1.931E-02
3290075	*FAM115B*	1.04	1.938E-02
610577	*LOC401676*	-1.20	1.940E-02
1240739	*LEPRE1*	-0.82	1.941E-02
360142	*RIPK3*	0.77	1.960E-02
2060047	*B4GALT5*	1.06	1.966E-02
4890092	*TSEN54*	-1.15	1.975E-02
2640441	*PRAGMIN*	-0.87	1.975E-02
4010494	*UBN2*	0.91	2.078E-02
240333	*ETS1*	-0.98	2.085E-02
1240450	*CD27*	-1.19	2.085E-02
4390576	*DFFA*	0.88	2.100E-02
4060446	*LOC649150*	-0.97	2.104E-02
7550161	*ADK*	-0.93	2.120E-02
510435	*PKIA*	-1.13	2.120E-02
4480446	*LOC654350*	-1.87	2.123E-02
1430347	*SLC7A6*	-0.90	2.131E-02
1940600	*LOC729839*	1.14	2.131E-02
940753	*RALB*	0.92	2.141E-02
7320731	*MAP3K4*	-0.99	2.145E-02
1070300	*OGT*	-0.90	2.170E-02
6840605	*ATM*	-1.01	2.182E-02

4850142	*KIAA1370*	-1.16	2.182E-02
2710646	*MSRB2*	1.03	2.228E-02
4050168	*Hs.526948*	0.86	2.228E-02
6660382	*CDKN1A*	1.23	2.246E-02
2450400	*RASGRP3*	1.68	2.276E-02
2120279	*CREBZF*	-1.29	2.276E-02
6940274	*PTGDR*	-1.37	2.276E-02
5360307	*LOC644937*	-0.79	2.284E-02
2480719	*NSMCE2*	-1.38	2.303E-02
430368	*ZNF211*	-0.93	2.304E-02
3420561	*RPA2*	-0.81	2.306E-02
4900639	*GGNBP2*	-0.88	2.312E-02
5270296	*TFB2M*	-0.90	2.315E-02
4850670	*EIF4E3*	0.80	2.320E-02
3140047	*CROP*	-1.49	2.341E-02
4070376	*NCRNA00219*	-0.97	2.341E-02
1050196	*PRKAG2*	1.04	2.341E-02
1260239	*ARHGAP27*	0.77	2.342E-02
5670682	*FLJ32255*	1.12	2.346E-02
2000367	*LOC647436*	-1.38	2.346E-02
1660246	*GNB4*	1.82	2.367E-02
7650091	*FNBP4*	-0.87	2.374E-02
2940687	*C9orf5*	-0.99	2.380E-02
620543	*SLC4A7*	-1.27	2.394E-02
6980639	*ANKFY1*	0.85	2.401E-02
4760433	*C16orf7*	1.09	2.401E-02
1440192	*LGALS9B*	1.12	2.401E-02
6350064	*LOC728658*	-0.90	2.418E-02
4890500	*TOR1A*	0.70	2.434E-02
3180541	*LOC440093*	0.95	2.439E-02
6290400	*CD247*	-0.82	2.454E-02
6380255	*RPS3A*	-1.73	2.456E-02
5270088	*ZMYM2*	-0.97	2.465E-02
7400487	*Hs.563552*	0.95	2.470E-02
990343	*SP4*	-0.98	2.475E-02
60619	*KLRF1*	-1.82	2.499E-02
7320551	*LYN*	0.77	2.507E-02
610554	*USP25*	1.20	2.512E-02
1820253	*PRKRIR*	-1.00	2.517E-02
3610576	*SP140L*	0.81	2.534E-02
4060669	*CCDC76*	-1.56	2.540E-02
2510082	*GCET2*	-0.96	2.545E-02
5080670	*GBA3*	1.03	2.553E-02
7210538	*SNORA43*	-0.93	2.560E-02
5960722	*TUSC4*	-0.91	2.563E-02
1450390	*RPL17*	-1.87	2.563E-02

4860161	*SP100*	1.01	2.563E-02
3710719	*MRPL45*	-0.77	2.580E-02
2810750	*LOC100134234*	0.81	2.619E-02
5340458	*NSUN5*	-0.86	2.635E-02
1300706	*MTHFD2*	1.02	2.639E-02
770164	*RPAIN*	-0.94	2.658E-02
2680561	*N4BP1*	0.98	2.671E-02
50075	*LOC100128274*	0.97	2.671E-02
2450707	*SIGLEC16*	1.16	2.671E-02
7330326	*WDR42A*	-0.77	2.686E-02
6520577	*DIP2A*	-1.21	2.693E-02
4640367	*SFI1*	-1.16	2.717E-02
5360093	*SECTM1*	0.93	2.720E-02
3890689	*CD247*	-0.77	2.720E-02
6660398	*FCN1*	0.74	2.720E-02
1510026	*FLVCR2*	0.78	2.720E-02
1990446	*KIAA0040*	0.91	2.725E-02
840037	*Hs.566482*	0.89	2.726E-02
2750039	*RBMS2*	1.20	2.736E-02
3390020	*MBTPS1*	-0.70	2.736E-02
2030360	*FAM159A*	-1.08	2.736E-02
4220259	*CTSZ*	1.14	2.745E-02
1400240	*LDHB*	-1.54	2.751E-02
6370470	*APOL1*	1.08	2.751E-02
5260403	*ST3GAL5*	0.73	2.757E-02
7510392	*DIP2B*	0.72	2.765E-02
2470541	*LOC642250*	-1.71	2.775E-02
1940050	*LOC388720*	-0.97	2.777E-02
3890747	*LOC158345*	-0.99	2.777E-02
7210484	*C6orf108*	-1.10	2.792E-02
4780678	*ZAP70*	-1.14	2.814E-02
6960411	*RCN2*	-1.08	2.834E-02
4490520	*EBI2*	-1.31	2.835E-02
2490341	*LOC100129742*	-1.71	2.843E-02
1570537	*TOP1*	0.93	2.850E-02
2060670	*LOC653689*	0.93	2.852E-02
4220053	*SKAP1*	-0.90	2.859E-02
7040731	*Hs.154336*	-1.09	2.860E-02
5360445	*LOC645693*	-1.11	2.873E-02
4890328	*SIDT2*	1.02	2.875E-02
3360446	*Hs.194225*	-0.87	2.875E-02
3780528	*RPS27A*	-1.42	2.879E-02
6330196	*MAL*	-0.95	2.879E-02
1470020	*GTPBP1*	0.90	2.879E-02
7380725	*GRB2*	0.80	2.887E-02
2060411	*LOC652616*	1.60	2.887E-02

430315	*TUBGCP5*	-0.78	2.909E-02
580066	*C1GALT1*	1.26	2.918E-02
3180438	*RPS3A*	-1.46	2.956E-02
5550504	*LOC642808*	1.07	2.974E-02
60358	*LOC387930*	-0.98	2.974E-02
160070	*C16orf30*	-1.20	2.988E-02
4890615	*RPL6*	-1.08	2.988E-02
6940386	*PHF14*	-0.94	2.998E-02
2140524	*HIST1H3D*	1.24	2.998E-02
2350066	*HLA-A*	0.84	3.022E-02
6180088	*ATP6V0E2*	-0.85	3.031E-02
4280129	*ELP2*	-0.83	3.039E-02
6650164	*LOC400027*	-0.91	3.039E-02
3870292	*ZNF398*	-0.79	3.039E-02
2850600	*TMEM204*	-1.11	3.045E-02
2710561	*FLJ44790*	0.82	3.046E-02
6420707	*IL8RBP*	1.33	3.046E-02
2360494	*Hs.485155*	-0.75	3.046E-02
6770673	*SOCS2*	-0.86	3.046E-02
270215	*ARSB*	0.98	3.063E-02
1940162	*GBP2*	1.11	3.079E-02
670463	*AK3*	-0.92	3.085E-02
1430288	*LOC100129668*	0.97	3.097E-02
2710358	*ENOSF1*	-1.25	3.117E-02
1300142	*NCRNA00153*	-1.02	3.117E-02
3060278	*MGC15763*	-1.11	3.117E-02
6270040	*ZNF831*	-0.98	3.139E-02
1780209	*LOC645378*	-0.89	3.166E-02
3460537	*LOC100132488*	-1.09	3.166E-02
3990224	*TNFRSF25*	-1.07	3.180E-02
6100064	*CRYGS*	-1.00	3.184E-02
6770605	*LOC647135*	1.08	3.184E-02
5720703	*AKTIP*	-0.91	3.184E-02
6560411	*C9orf66*	0.98	3.192E-02
5560364	*OSTalpha*	0.87	3.194E-02
380446	*FLJ44674*	0.96	3.217E-02
620692	*SLC25A42*	-0.97	3.221E-02
6510397	*WDR19*	-0.83	3.229E-02
2490670	*GNPTAB*	-0.82	3.240E-02
6330408	*LOC388339*	-1.13	3.240E-02
3940438	*NCF1*	1.51	3.243E-02
4480386	*LOC642660*	0.96	3.255E-02
3450154	*TRAF3IP3*	-0.90	3.259E-02
4850092	*ACSM5*	0.82	3.268E-02
2370041	*LRRN3*	-1.64	3.280E-02
6330538	*MAD2L1BP*	0.89	3.280E-02

2470240	*C1orf38*	0.70	3.287E-02
5310735	*EIF3L*	-0.61	3.287E-02
4050327	*LOC284701*	0.94	3.287E-02
2230561	*LOC144383*	1.31	3.308E-02
940450	*AMT*	-0.96	3.311E-02
4780196	*CD300A*	0.83	3.311E-02
2760544	*PBX4*	-0.86	3.334E-02
3930326	*LOC728014*	-0.97	3.334E-02
3400612	*WDFY1*	1.11	3.334E-02
4610484	*C14orf93*	0.75	3.346E-02
5550592	*LOC728661*	-0.69	3.348E-02
160091	*LOC399804*	-1.38	3.392E-02
6200019	*KLRB1*	-1.70	3.411E-02
3460592	*FEZF2*	0.93	3.411E-02
3840215	*FAM102A*	-1.06	3.436E-02
2230026	*KLF5*	0.96	3.436E-02
6900601	*Hs.157344*	1.13	3.439E-02
2710093	*ZNF74*	-1.04	3.439E-02
1170176	*CAMK2G*	-0.94	3.439E-02
1430131	*LOC652904*	0.85	3.459E-02
4040037	*EBI2*	-1.33	3.549E-02
3310341	*FYB*	1.07	3.549E-02
630369	*LOC100127993*	-0.73	3.549E-02
3420300	*EEF2K*	-0.70	3.549E-02
7000270	*PYHIN1*	-1.13	3.555E-02
270240	*SLC26A8*	1.29	3.578E-02
6940465	*RALGAPA1*	-0.82	3.578E-02
3940220	*RPS27*	-1.13	3.593E-02
6400132	*MEF2A*	1.21	3.598E-02
4590446	*MSL3*	0.68	3.604E-02
4070168	*METAP1*	-0.75	3.604E-02
1510088	*ATP1B3*	0.75	3.604E-02
5690202	*AKIRIN2*	0.86	3.604E-02
1740142	*LOC647074*	-0.77	3.613E-02
1690039	*PRKCE*	1.27	3.613E-02
510064	*DIP2B*	0.77	3.615E-02
3130403	*LOC643779*	-0.99	3.618E-02
110719	*SLC16A3*	0.87	3.638E-02
6770017	*KLF12*	-1.06	3.651E-02
5910148	*ACYP1*	-1.20	3.651E-02
6290681	*TOMM20*	-0.83	3.677E-02
1450600	*GOLGA6B*	-1.44	3.677E-02
3450326	*NAGK*	1.04	3.677E-02
6180022	*PCM1*	-1.06	3.677E-02
3610092	*RBM23*	0.67	3.677E-02
4200040	*Hs.529633*	0.79	3.677E-02

1230026	*LOC339192*	-0.82	3.677E-02
730528	*PLAUR*	1.22	3.677E-02
3170452	*ORMDL1*	-0.90	3.691E-02
7550639	*GNPDA2*	-0.95	3.691E-02
4610672	*FLJ14213*	-0.92	3.701E-02
5090424	*RPS27*	-1.18	3.710E-02
3610053	*LOC100128936*	-0.97	3.729E-02
2570368	*LOC100129158*	-0.92	3.737E-02
4250156	*EBP*	-1.04	3.777E-02
4070180	*PRRT3*	-0.91	3.781E-02
1940021	*GRN*	1.03	3.784E-02
7040168	*TTC19*	-0.86	3.784E-02
4220431	*EXT1*	0.92	3.784E-02
2940739	*RASSF1*	-0.92	3.803E-02
5670497	*LOC644363*	-0.96	3.803E-02
3400364	*LOC647766*	0.92	3.803E-02
130670	*ZDHHC5*	0.70	3.814E-02
4150397	*HELQ*	-0.79	3.814E-02
5900129	*CROP*	-1.23	3.814E-02
1430360	*ABHD14A*	-1.03	3.814E-02
2810601	*LEF1*	-0.98	3.814E-02
3170703	*LY9*	-0.93	3.816E-02
3130382	*LOC730861*	0.96	3.830E-02
1820619	*LOC388122*	-0.89	3.844E-02
2650564	*RARRES3*	0.97	3.858E-02
6510010	*LOC730631*	0.98	3.860E-02
2470020	*TNPO1*	-0.85	3.862E-02
4540398	*BEND5*	-0.99	3.869E-02
20164	*YIPF1*	0.62	3.884E-02
6580017	*LOC100132742*	-1.83	3.908E-02
1050082	*KIAA1147*	-0.96	3.908E-02
1710639	*RBM4B*	-0.69	3.909E-02
4180333	*C6orf165*	0.86	3.910E-02
5560131	*ATOX1*	0.91	3.910E-02
6520273	*LOC646527*	-1.73	3.910E-02
2710754	*CD96*	-1.06	3.910E-02
730673	*PCMTD2*	-1.36	3.910E-02
1170309	*LOC642357*	-0.78	3.939E-02
2140706	*NARG1L*	-1.18	3.952E-02
2570739	*Hs.538367*	0.92	3.971E-02
4230097	*HMGB1*	-1.01	3.971E-02
4010768	*CTLA4*	-1.09	3.972E-02
3060458	*FAM111A*	0.91	3.974E-02
5720537	*ACPL2*	-0.91	3.977E-02
4610681	*LOC653658*	-1.53	3.977E-02
2480403	*LOC285900*	-1.19	3.984E-02

580575	*LOC255620*	1.19	3.987E-02
160291	*MVP*	0.89	3.987E-02
1030025	*PTP4A1*	0.78	3.995E-02
3130370	*ZNF83*	-1.49	4.044E-02
5220068	*C4orf14*	-0.69	4.062E-02
630035	*LOC645296*	-1.59	4.062E-02
6040114	*PPP2R4*	0.71	4.067E-02
770424	*CTGLF3*	-0.77	4.067E-02
2710292	*H2AFZ*	-0.82	4.078E-02
2230113	*TANK*	0.73	4.128E-02
150112	*LOC100133931*	-1.54	4.129E-02
1010048	*CXXC5*	-0.96	4.130E-02
5910164	*LARP1B*	-1.15	4.151E-02
5670465	*ADM*	1.28	4.151E-02
6650672	*ZNF439*	-1.14	4.161E-02
2570187	*ZNF30*	-1.18	4.166E-02
4760538	*ZC3H3*	0.70	4.166E-02
6290546	*PURB*	-0.78	4.170E-02
5820470	*TBC1D26*	0.94	4.187E-02
2480056	*LOC340260*	-0.89	4.241E-02
3060187	*LOC100133923*	-1.30	4.246E-02
290180	*LOC647030*	-1.59	4.283E-02
1300369	*LOC255783*	0.88	4.310E-02
2760491	*ATG16L1*	-0.61	4.311E-02
1090292	*BMI1*	-1.00	4.320E-02
1820093	*HNRPA1P4*	-1.38	4.349E-02
5870682	*Hs.537742*	1.03	4.349E-02
2470270	*DUS4L*	-0.77	4.353E-02
7610390	*NOD1*	1.00	4.365E-02
4250008	*ATM*	-0.97	4.374E-02
2000100	*ABI2*	0.90	4.389E-02
6770168	*CRTAP*	-0.88	4.389E-02
6590669	*RAB24*	0.98	4.390E-02
2450280	*ZCCHC17*	-0.84	4.390E-02
5570180	*LCK*	-1.01	4.413E-02
3180382	*FKBP15*	0.73	4.427E-02
2470142	*ZFP82*	-1.10	4.449E-02
3130324	*LOC389404*	-1.53	4.449E-02
3710202	*MST1*	-0.90	4.449E-02
5490341	*CLEC2D*	-1.16	4.459E-02
10433	*ITGA10*	0.77	4.461E-02
2680722	*HIF1A*	0.89	4.495E-02
6860382	*OGFR*	1.02	4.527E-02
2120703	*LOC645173*	-0.86	4.527E-02
940564	*LOC642780*	0.78	4.538E-02
4390167	*LOC100129553*	-0.92	4.542E-02

5080482	*TNPO1*	-0.68	4.543E-02
1980520	*RPL6*	-0.84	4.550E-02
6550703	*WSB2*	0.70	4.558E-02
360731	*LOC728661*	-0.80	4.560E-02
830403	*PSME2*	1.33	4.566E-02
1820274	*HERC1*	-0.71	4.582E-02
2630161	*NOL11*	-0.83	4.584E-02
990128	*MBNL2*	-1.25	4.584E-02
7050328	*SEMA4C*	-0.84	4.584E-02
510762	*FAM62B*	-0.61	4.584E-02
4760053	*C2orf89*	-1.06	4.596E-02
290750	*SAMD3*	-1.13	4.623E-02
7380152	*SLC35E3*	-0.75	4.660E-02
3420128	*AP3M2*	-1.03	4.679E-02
7000059	*LOC642828*	-0.95	4.679E-02
7550215	*FCGR2B*	1.12	4.723E-02
450433	*LOC100129067*	-1.38	4.723E-02
580717	*LOC441246*	-1.17	4.735E-02
5570730	*TICAM1*	0.73	4.774E-02
2320653	*ECHDC2*	-0.91	4.813E-02
4010270	*LOC440731*	1.04	4.826E-02
1410020	*TMCO3*	-0.98	4.826E-02
2470017	*LILRB3*	0.84	4.827E-02
1340241	*C5*	1.12	4.862E-02
3170020	*DGKA*	-0.95	4.866E-02
5270315	*WDR12*	-0.76	4.870E-02
1230630	*PITPNC1*	-0.79	4.870E-02
5570634	*LOC645693*	-1.30	4.870E-02
5050541	*LOC653232*	-0.76	4.870E-02
7050451	*LOC100134504*	-1.86	4.877E-02
4200543	*GPX7*	-0.85	4.891E-02
5820129	*PABPC1*	-0.55	4.894E-02
4480280	*BRPF3*	-0.68	4.904E-02
2320139	*RPS27A*	-0.90	4.938E-02
5290523	*ARHGAP25*	1.09	4.946E-02
4890156	*RCAN3*	-0.80	4.956E-02
6840348	*BRI3BP*	-0.72	4.956E-02
5490524	*ZNF302*	-1.47	4.956E-02
7510379	*SACS*	-1.61	4.956E-02
1410035	*RPS6*	-1.18	4.956E-02
4040026	*LOC728590*	-1.40	4.956E-02
3780167	*ZNF200*	0.83	4.956E-02
130131	*ACSL4*	1.13	4.956E-02
4150100	*PASK*	-0.93	4.956E-02
990014	*C22orf32*	-0.91	4.957E-02
7650639	*CHRNB1*	0.76	4.962E-02

6620438	*TOP1P1*	0.69	4.962E-02
2650026	*LOC402694*	-0.66	4.969E-02
4640689	*EIF4A2*	-0.88	4.975E-02
1430176	*OGT*	-1.04	4.991E-02

References

1. Lillie PJ, Berthoud TK, Powell TJ, Lambe T, Mullarkey C, Spencer AJ, Hamill M, Peng Y, Blais ME, Duncan CJ, Sheehy SH, Havelock T, Faust SN, Williams RL, Gilbert A, Oxford J, Dong T, Hill AV & Gilbert SC. (2012) Preliminary assessment of the efficacy of a T-cell-based influenza vaccine, MVA-NP+M1, in humans. Clin Infect Dis 55*:*19-25.

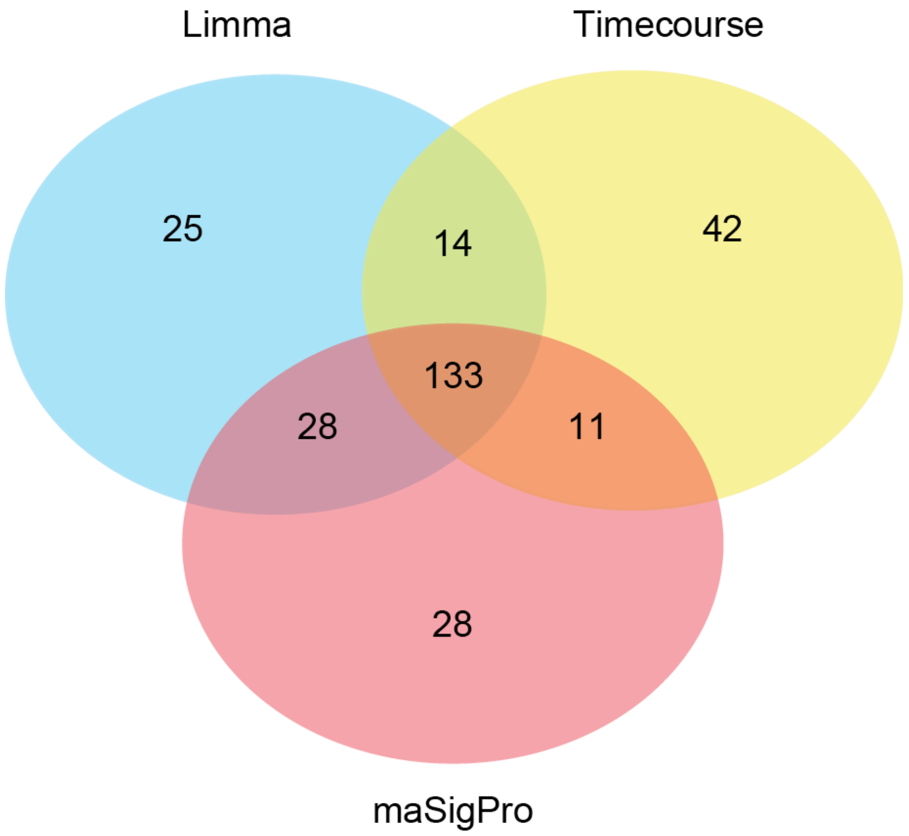

Fig. S1 Comparison of methods for determining differential expression of probes. Three R packages (limma, maSigPro and timecourse) were used to determine the differentially expressed probes between the four moderate/severe LCI samples 48 hours post-challenge and the remaining samples. The top 200 most significant probes from each analysis were used to compare the overlap between methods.

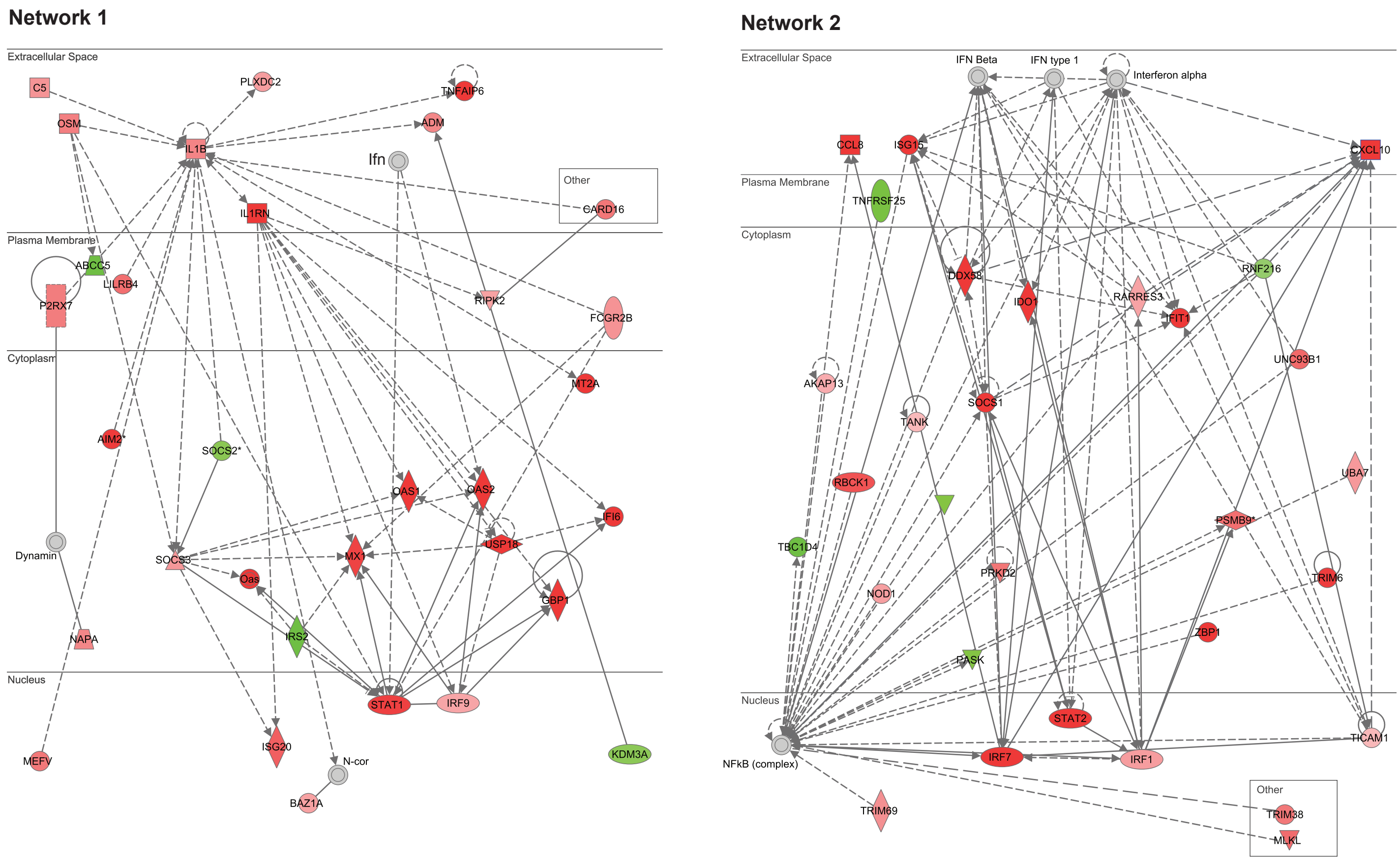

Fig. S2 Gene networks identified in differentially expressed genes at 48 hour post challenge between four subjects with moderate/severe LCI vs remaining subjects. The top two gene networks identified in IPA are shown in the context of subcellular localisation. Both networks involve antimicrobial/inflammatory response and IFN signalling.

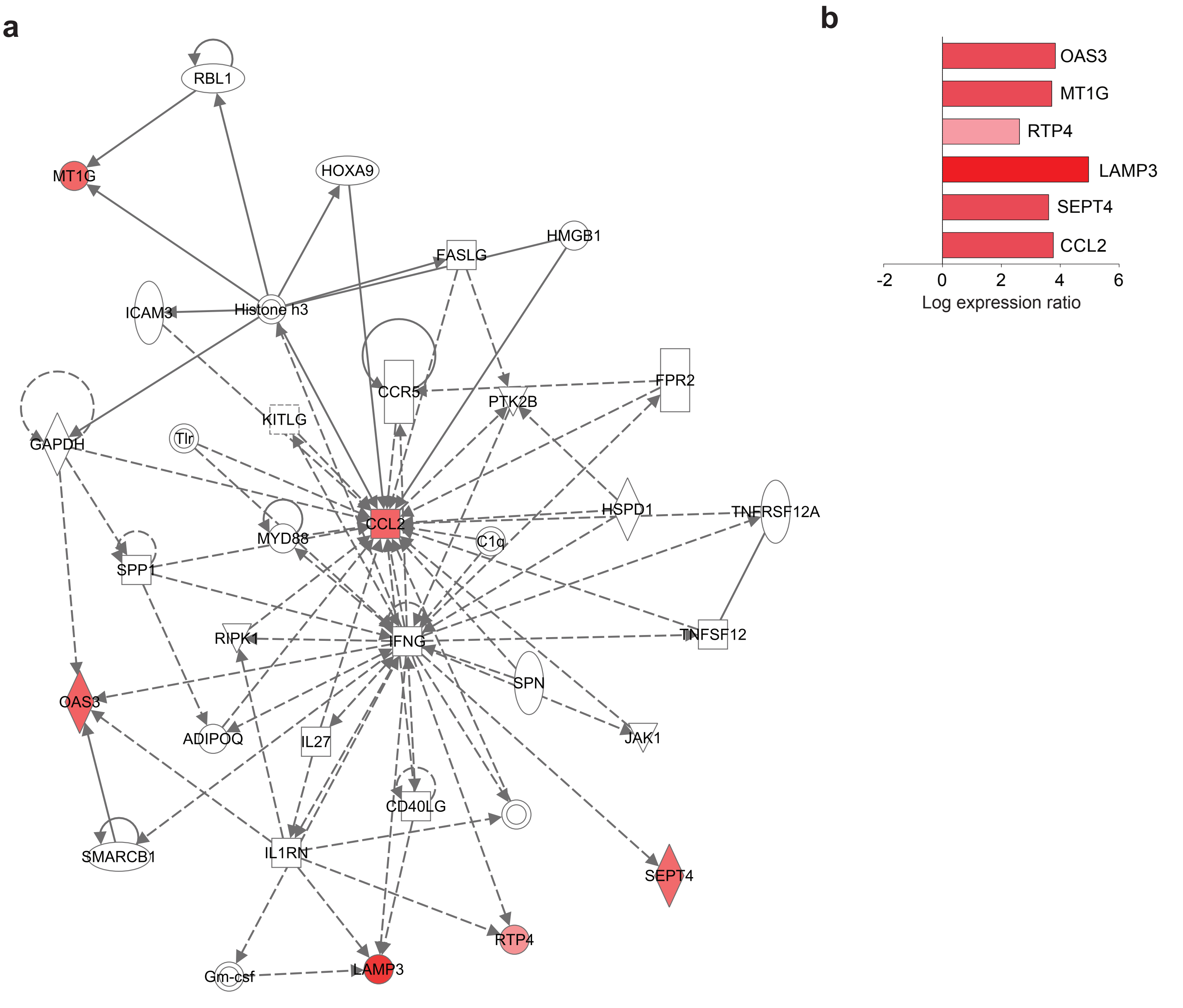

Fig. S3. Network analysis of six gene set. To explore the possible relationships between the six gene predictive set a network analysis was performed using IPA. **a**. Results of network analysis. A single gene network is found that includes all six genes when these six genes are analysed. The most significant predicted upstream regulator for these six genes is IFNγ (p = 3.0x10-9). **b**. Differential gene expression between four individuals with moderate/severe LCI vs other individuals at 48 hours post-challenge. Log expression ratios are shown with upregulation of all six genes in individuals with moderate/severe LCI observed.
